# Diversity and distribution of nuclease bacteriocins in bacterial genomes revealed using Hidden Markov Models

**DOI:** 10.1371/journal.pcbi.1005652

**Published:** 2017-07-17

**Authors:** Connor Sharp, James Bray, Nicholas G. Housden, Martin C. J. Maiden, Colin Kleanthous

**Affiliations:** 1 Department of Biochemistry, University of Oxford, Oxford, United Kingdom; 2 Department of Zoology, University of Oxford, Oxford, United Kingdom; University College London, UNITED KINGDOM

## Abstract

Bacteria exploit an arsenal of antimicrobial peptides and proteins to compete with each other. Three main competition systems have been described: type six secretion systems (T6SS); contact dependent inhibition (CDI); and bacteriocins. Unlike T6SS and CDI systems, bacteriocins do not require contact between bacteria but are diffusible toxins released into the environment. Identified almost a century ago, our understanding of bacteriocin distribution and prevalence in bacterial populations remains poor. In the case of protein bacteriocins, this is because of high levels of sequence diversity and difficulties in distinguishing their killing domains from those of other competition systems. Here, we develop a robust bioinformatics pipeline exploiting Hidden Markov Models for the identification of nuclease bacteriocins (NBs) in bacteria of which, to-date, only a handful are known. NBs are large (>60 kDa) toxins that target nucleic acids (DNA, tRNA or rRNA) in the cytoplasm of susceptible bacteria, usually closely related to the producing organism. We identified >3000 NB genes located on plasmids or on the chromosome from 53 bacterial species distributed across different ecological niches, including human, animals, plants, and the environment. A newly identified NB predicted to be specific for *Pseudomonas aeruginosa* (pyocin Sn) was produced and shown to kill *P*. *aeruginosa* thereby validating our pipeline. Intriguingly, while the genes encoding the machinery needed for NB translocation across the cell envelope are widespread in Gram-negative bacteria, NBs are found exclusively in γ-proteobacteria. Similarity network analysis demonstrated that NBs fall into eight groups each with a distinct arrangement of protein domains involved in import. The only structural feature conserved across all groups was a sequence motif critical for cell-killing that is generally not found in bacteriocins targeting the periplasm, implying a specific role in translocating the nuclease to the cytoplasm. Finally, we demonstrate a significant association between nuclease colicins, NBs specific for *Escherichia coli*, and virulence factors, suggesting NBs play a role in infection processes, most likely by enabling pathogens to outcompete commensal bacteria.

## Author summary

Bacteria deploy a variety of antimicrobials to kill competing bacteria. Nuclease bacteriocins are a miscellaneous group of protein toxins that target closely related species, cleaving nucleic acids in the cytoplasm. It has proved difficult to establish how widespread bacteriocins are in bacterial populations due to the high diversity of bacteriocin-encoding genes. Here, we describe an *in silico* approach to identify nuclease bacteriocin genes in bacterial genomes and to distinguish them from other competition toxins. Bacteria that contain nuclease bacteriocin genes are found in many different types of environment but are prevalent in niches where interbacterial competition is likely to be high. Nuclease bacteriocins are found exclusively in *γ*-proteobacteria and are particularly abundant in the *Enterobacteriaceae* and *Pseudomonadaceae* families. Although the sequences we identify are indeed diverse (<20% sequence identity between protein families) we show that all nuclease bacteriocins contain an invariant motif, usually within a common structural scaffold, that is implicated in translocating the cytotoxic nuclease to the cytoplasm. Finally, we show that nuclease bacteriocins in pathogenic *E*. *coli* are strongly associated with virulence factors suggesting they play a role in pathogenicity mechanisms.

## Introduction

Bacteriocins were the first of the interbacterial competition systems to be discovered [1]. They take the form of antimicrobial peptides or proteins released to the environment that have activity against closely related organisms. Experimental and theoretical work has suggested that bacteriocins are important agents of competition between microbial communities [2–6]. The potent antimicrobial activity of bacteriocins has generated interest in their application in agriculture [7], the food industry [8], and as potential clinical therapies for bacterial infections [9, 10]; however, without understanding the distribution of bacteriocins, the significance of their role in bacterial competition remains uncertain and their exploitation consequently limited. Here, we circumvent one of the problems in identifying protein bacteriocins in bacterial genomes, their sequence and domain diversity, by developing a bioinformatics pipeline that identifies enzymatic nuclease bacteriocins (NBs). NBs are one of the major classes of protein bacteriocins. The new tools we have developed allowed us to determine NB species distribution and evaluate current ideas as to how such molecules are deployed by Gram-negative bacteria.

The best studied of the Gram-negative protein bacteriocins are the colicins produced by *E*. *coli*. Colicin gene expression is typically coupled to the SOS stress response and nutrient status of the cell [11, 12]. Despite varying in size, colicins have a common tripartite structure, composed of an N-terminal translocation (T-) domain, a central receptor binding (R-) domain and a C-terminal cytotoxic domain. Colicins deliver one of five cytotoxic activities into cells; a pore-forming ionophore or lipid II hydrolase are delivered to the periplasm whereas DNases, tRNases or rRNases are delivered to the cytoplasm [13]. Colicin-producing bacteria protect themselves through the action of a small immunity protein that in the case of nuclease colicins is co-expressed and released from cells bound tightly to its colicin [14–16]. Colicins enter a target cell by binding an outer membrane receptor, typically an iron or vitamin transporter, and then parasitizing another outer membrane protein as a translocation portal for its T-domain to contact one of two trans-periplasmic proton motive force (pmf)-coupled systems: Tol for group A (e.g. ColE9) or Ton for group B (e.g. ColD) colicins [13]. The immunity proteins of nuclease colicins are dissociated at the cell surface in a pmf-dependent step during translocation [17]. Colicin-like NBs have been identified in pathogens such as *Klebsiella pneumoniae* (klebicins) and *Pseudomonas aeruginosa* (pyocins), all having a distinct multi-domain architecture and associated immunity protein [18–20].

Colicins (and colicin-like proteins) are thought to play a role in the co-existence of mixed microbial communities, and possibly as virulence determinants during proliferation within such communities [2, 21]. ExPEC bacteria, for example, show a significant association between virulence factors and colicins in *E*. *coli* isolates that cause bacteraemia [22, 23]. In a mouse gut infection model, colicin production by *Salmonella enterica* serovar Typhimurium is exploited by the organism to outcompete commensal *E*. *coli* during inflammation [24]. A recent survey of *Shigella sonnei* isolates in Vietnam spanning three decades identified a colicin-producing plasmid as one of the factors that overcame a dispersal bottleneck, helping the organism become established in the population as the major cause of dysentery [25]. Hence identifying the ecological niches in which colicins and colicin-like proteins are found and ascertaining their prevalence amongst pathogens are missing pieces of the microbial ecology puzzle that has ramifications for clinical epidemiology, biotechnology and biomedicine.

Multiple factors make NB genes difficult targets for *in silico* identification. First, NBs are often (but not always) plasmid-encoded and only present in a fraction of isolates [26]. Second, extensive sequence diversification and domain rearrangements hamper identification of NBs. Finally, the nuclease domains of NBs are similar to the cytotoxic domains of other polymorphic toxin families, such as contact-independent inhibition (CDI) systems, further complicating their identification. In this study, we establish methods for the identification of NBs from large bacterial genome datasets. To overcome the low sequence identity between NBs, we use profile hidden markov models to identify conserved motifs in NB cytotoxic domains and their associated immunity proteins. We find that NBs are present at differing levels of abundance in bacterial species and that organisms exploit them in many varied ecological niches. Our analysis, validated for a novel NB shown to be active against *P*. *aeruginosa*, reveals the extent of domain rearrangements and sequence diversification that has occurred in NB genes, identifies a critical motif implicated in NB translocation and associates NBs in bacterial pathogenesis.

## Results and discussion

### Constructing Hidden Markov Models for the identification of NBs in bacterial genomes

**Fig 1:**
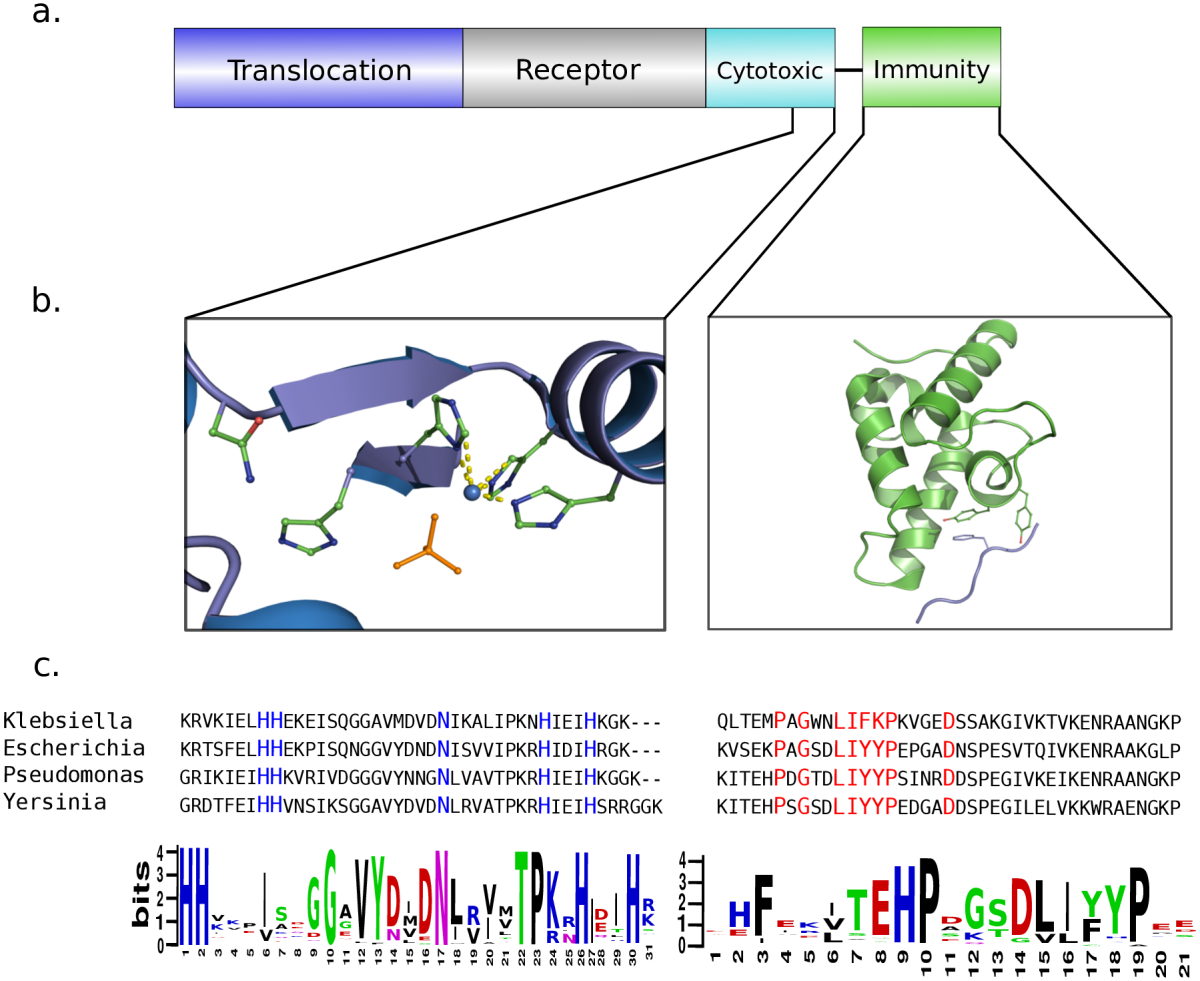
Identifying genetically linked conserved cytotoxic and immunity motifs is a powerful and accurate way to identify NB operons. ***a***, Gene/protein organisation of a typical nuclease bacteriocin from *E*. *coli*. ***b***, *Left-hand panel*, key interactions of conserved catalytic residues of the HNH motif of DNase bacteriocins. The two histidine residues of the HNH motif are involved in coordinating a divalent metal ion and the asparagine constrains the metal binding loop. The phosphate anion denotes the position of the scissile phosphate in substrate DNA (PDB code, 1V14 [34]). *Right-hand panel*, the helical immunity protein (*green*) showing the conserved aromatic residues of the α-helix III, which forms a critical part of the binding site for the DNase domain [15]. ***c***, Conserved residues used to form the HMM profile of each protein are highlighted in the sequence alignments.

Protein bacteriocins are difficult to identify in bacterial genomes using conventional pairwise alignment strategies. We therefore developed a bioinformatics pipeline to identify bacteriocins using profile Hidden Markov models (HMMer 3.1) [27] centred on the catalytic regions of the nuclease cytotoxic domain of the bacteriocin. Pore-forming bacteriocins were excluded from the study due to the absence of similarly conserved motifs. Five different NB families have been described in the literature, two DNases and three RNases (Fig 1 and S1 Fig) and the profile pairs were based on conserved motifs identified in their catalytic centres. For example, HNH-type endonuclease bacteriocins have a 30-residue motif (also referred to as the ββα-Me motif) as their active site (HHX_14_NX_8_HX_3_H) (Fig 1). Conserved motifs are similarly identifiable in the cytotoxic domains of non-HNH DNase, tRNase and rRNase NBs [28, 29] (S1 Fig). These motifs are however frequently found in other bacterial contexts; for example, the HNH motif is also found in Type II restriction and homing endonucleases, mismatch repair enzymes, and is the catalytic core of the CRISPR-Cas9 complex [30–32]. Hence three additional criteria were used to distinguish NBs from other systems and to validate them as *bona fide* NBs. First, the presence of conserved motifs within the adjoining immunity protein. Immunity proteins are specific, tight-binding inhibitors of bacteriocins that protect the producing cell from the action of its own toxin [16]. In the case of NBs, immunity genes are co-expressed with the bacteriocin. The resulting heterodimeric complex is often released from the cell through the action of a bacteriocin-release protein or lysis protein, which is often encoded within bacteriocin operons [13]. In cases where a bacteriocin-release protein has not been identified latent prophage genes have been implicated in release [33]. NB-specific immunity proteins are only jettisoned during translocation of the toxin into a susceptible cell [17]. To increase the specificity of our searches we exploited the highly conserved genetic link between NB genes and their cognate immunity genes, creating ‘profile pairs’ where the immunity profile is within 60bps (or 200bps in the case of ColE5/pyocinS4 tRNase) downstream of the cytotoxic profile (S1 Table, S1 and S2 Figs). Second, the length of the NB sequence. NBs need multiple domains to translocate across the two membranes of the cell envelope and are typically >60 kDa in size. To be conservative, we filtered by sequence length, accepting sequences between 350–950 residues. Third, putative bacteriocins and the genes at the N-terminus are analysed for the presence of domains readily identifiable through the PFAM database as linked to other polymorphic toxin systems are excluded; for example, TSS6 effector proteins, which are often NB-related nucleases, or hemagglutinin repeats that are associated with contact dependent inhibitors.

We subjected 100,154 PubMLST Multispecies isolate database, covering >3,000 species, to our pipeline and identified 3094 bacteriocin genes in 2479 isolates across 53 species (S3 Fig, S2 Table). The largest non-bacteriocin contaminants of the database were uropathogenic-specific protein (*usp*) from *E*. *coli* and various short DNase containing proteins predicted to be effectors of the T6SS. *E*. *coli usp* has 40–50% sequence identity to the HNH DNase cytotoxic domain of NBs and was identified in over 2000 *E*. *coli* and *Salmonella enterica* genomes [35]. They were not included in our analysis as Usp has been shown to have genotoxic effects towards mammalian not bacterial cells and also contain an N-terminal domain of unknown function (DUF769), predicted to be a member of the Hcp-1 proteins of T6SS [35, 36]. A group of HNH containing nucleases were also located in the Gram-positive *Bacillus* genera which are linked to the SUKH (Syd, US22, Knr4 homology) domain immunity protein superfamily that bind to a variety of bacterial toxins of diverse nuclease and nucleic acid deaminase families [37]. The HNH motif was dissimilar to the NB DNase motif, having an extended linker region between the final histidine residues, HHX_14_N_8_HX_8_H. A non-HNH DNase T6SS effector was present in over half of *Klebsiella pneumoniae* strains. Other polymorphic toxin systems that share similar cytotoxic domains with NBs include the MAF proteins of *Neisseria* and a family of large T6SS effectors in the *Vibrio* genus that were identified by a LysM domain (PFAM 01476) and a recently described motif associated with type VI secretion, the MIX domain [38, 39]. Interestingly, we identified several bacterial species that utilize NBs as well as other competition systems such as T6SS, including *Pseudomonas aeruginosa*, *Escherichia coli* and *Yersinia pseudotuberculosis*. The dynamics of bacteria could utilise both diffusible and contact dependant competition systems is still unknown.

### NBs are found in a variety of environments but are exclusive to γ-proteobacteria

We next looked for an association between NBs across different environmental niches. The PATRIC database contains 6256 genomes with associated environmental data [40]. The >1500 types of listed environment were grouped by an automated keyword search using custom python scripts. 120 unique environments were identified across 239 NB containing bacteria. Whilst it is not surprising that the majority of NB containing *E*. *coli* were identified in the GI tract and the majority of *Pseudomonas* species associated with either soil or plant based environments, we also observed these bacteria in the invasive environments of blood and urine including patients suffering septicemia (*P*. *aeruginosa* VRFPA01 and *P*. *aeruginosa* VRFPA02) or urinary tract infection (*E*. *coli* UMEA-3342-1) (S4 Fig).

NBs are found in significant proportions in the *Enterobacteriaceae* and *Pseudomonadaceae* families, however the proportion of a species that contained NB genes varied greatly; <5% *E*. *coli* strains encoded NBs compared to 85% for *Pseudomonas aeruginosa* and 31% for *Klebsiella pneumoniae*, which agrees with previous experimental work [41–43]. A prevalent feature of *Pseudomonas* and *Klebsiella* isolates is their possession of multiple NBs, which is likely to be a factor in NB evolution through increased rates of recombination, as has been proposed for pore-forming bacteriocins [41]. *Klebsiella pneumoniae* genomes commonly encode a DNase bacteriocin as well as cloacin, a rRNase bacteriocin. This trend has also been observed in *Pseudomonas* species [44]. A similar distribution is observed with *Yersinia mollaretii* genomes where 4 isolates contained both DNase and rRNase NBs.

An important aspect of bacteriocin biology is how bacterial genomes encode these toxic molecules since this will have an impact on their distribution in bacterial populations. NBs of *E*. *coli* (colicins) are known to be plasmid encoded whereas NBs of *P*. *aeruginosa* (pyocins) are located on the chromosome [13]. As part of our analysis we instigated a bioinformatics protocol for establishing whether NBs were chromosomal or plasmid-encoded as this has not been shown for many species containing NB-encoding genes. Distinguishing these gene locations is, however, challenging due to the differing levels of assembly of the genomes interrogated by our pipeline. Using the Plasmid finder database and the Carattoli typing scheme [45] we could identify plasmid replicons within NB containing contigs. Addition of a conserved region identified for pColE1 replicons [46] allowed the typing of additional plasmids including large tRNase containing plasmids in *E*. *coli* (S5a Fig). To demonstrate plasmid or chromosomal association of contigs containing NB genes for species not covered within this typing scheme, that could not be typed using the Plasmid Finder database, a python script was implemented to measure the percentage alignment of a contig to a database of known plasmids. As anticipated, NBs from *Escherichia* and *Pseudomonas* exhibited a strong association with plasmid and chromosomes, respectively (S5b Fig). NBs from *Yersinia* species, however, demonstrated little or no alignment to plasmid sequences, suggesting these are chromosomally-located. Interestingly, species from *Enterobacter* displayed a bimodal distribution suggesting their NBs could be plasmid encoded or chromosomal. For a small number of NBs, these predictions were confirmed using NCBI fully assembled genomes; both plasmid and chromosomal NB genes were identified for *Enterobacter* isolates using this approach. In total, chromosomal NBs were identified in four genera: *Pseudomonas*, *Serratia*, *Yersinia and Enterobacter*. In the fully assembled genome of *Klebsiella pneumoniae* KPNIH12, plasmid transfer genes and an NB gene (klebicin B) were incorporated into the genome and associated with a putative IS903 transposase. NBs with high sequence identity to that of *Enterobacter cloacae* (cloacin DF13; CloDF13) were identified in species across multiple genera. CloDF13 is a ~60 kDa NB that utilizes an rRNase cytotoxic domain. MAUVE 2.0 was used to identify a plasmid in seven species that included transfer and mobilisation genes with over 90% identity to pCloDF13, a broad range transmissible bacteriocinogenic plasmid [47]. pCloDF13 was identified in *Enterobacter cloacae*, *Enterobacter aerogenes*, *Escherichia coli*, *Klebsiella pneumoniae* and *Salmonella enterica*.

**Fig 2:**
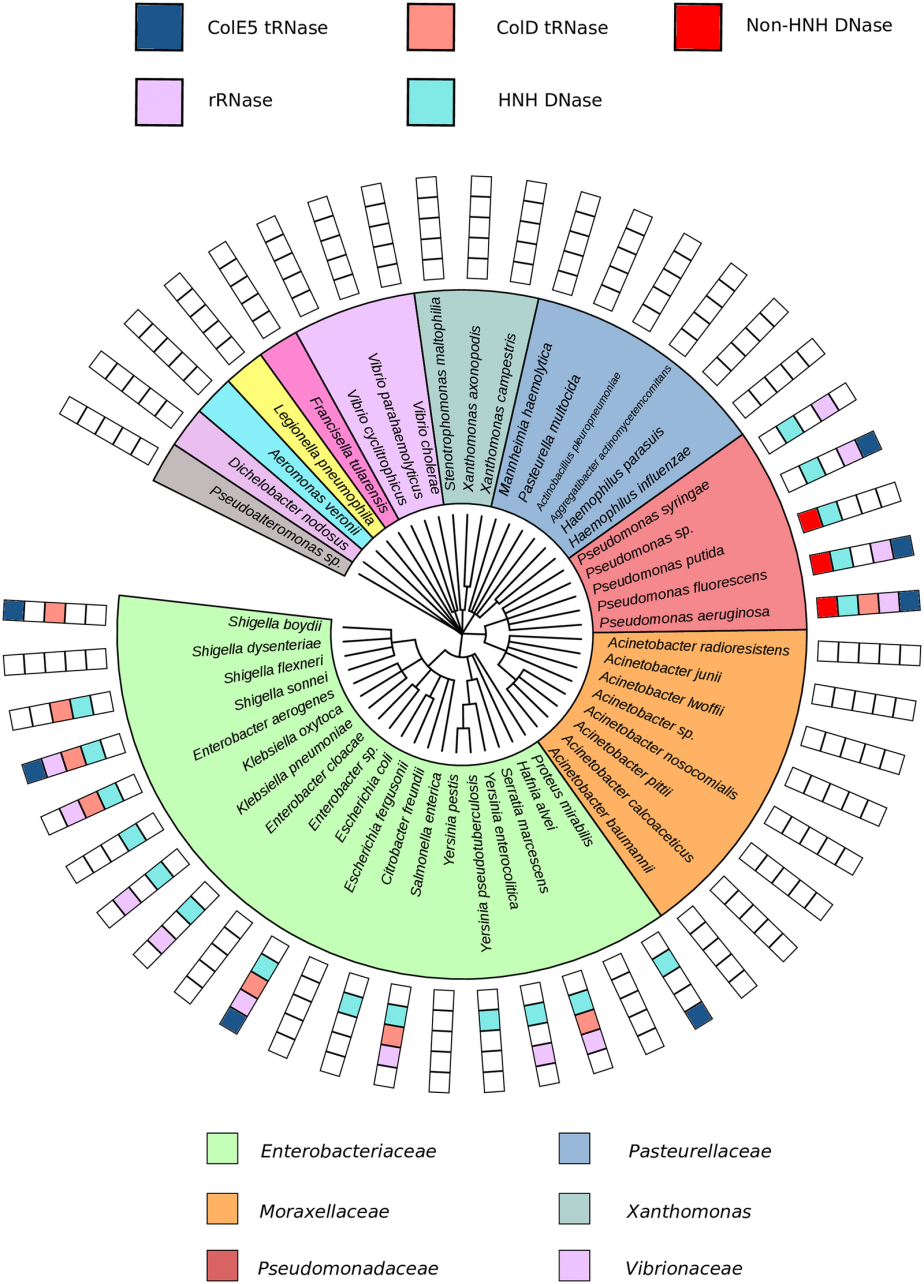
Distribution of NBs is restricted to the γ-proteobacteria. Taxonomic tree representing all γ-proteobacteria species in the pubMLST that have over 15 genomes, constructed using NCBI taxonomy commontree. The presence of different cytotoxic domains is indicated in the color key associated with each species. NBs are found throughout *Enterobacteriaceae* and *Pseudomonadaceae*.

A surprising conclusion of our analysis is that NBs are found exclusively in γ-proteobacteria (Fig 2, S6 Fig). Indeed, only one enzymatic bacteriocin has been identified outside of the γ-proteobacteria, a colicin M like bacteriocin in the β-proteobacterium *Burkholderia* [48]. We note, however, that unlike NBs which translocate to the bacterial cytoplasm to cleave nucleic acids, colicin M kills bacteria by cleaving the lipid II precursor of peptidoglycan within the periplasm [49]. In trying to rationalise why NBs appear only to be part of the antimicrobial armoury of γ-proteobacteria we considered two possibilities, the environment in which these organisms typically reside and the machinery involved in their uptake. Environment can be dismissed as a cause of this exclusivity since α-, β- and ε-proteobacteria live in many of the same environments as γ-proteobacteria that contain NB genes; for example, NBs are abundant in soil dwelling γ-proteobacteria yet α-, β- and δ-proteobacteria are all more prevalent in soil [50]. The translocation machinery hijacked by NBs, the Ton and Tol systems, are found beyond the γ-proteobacteria (defined using a profile HMM strategy; S3 Table, S7 Fig), which suggests that the mechanism of NB import is also not a limiting factor in their species distribution. It remains unclear why NBs are exclusively found in γ-proteobacteria.

### Sequence diversity and domain organisation of NBs

**Fig 3:**
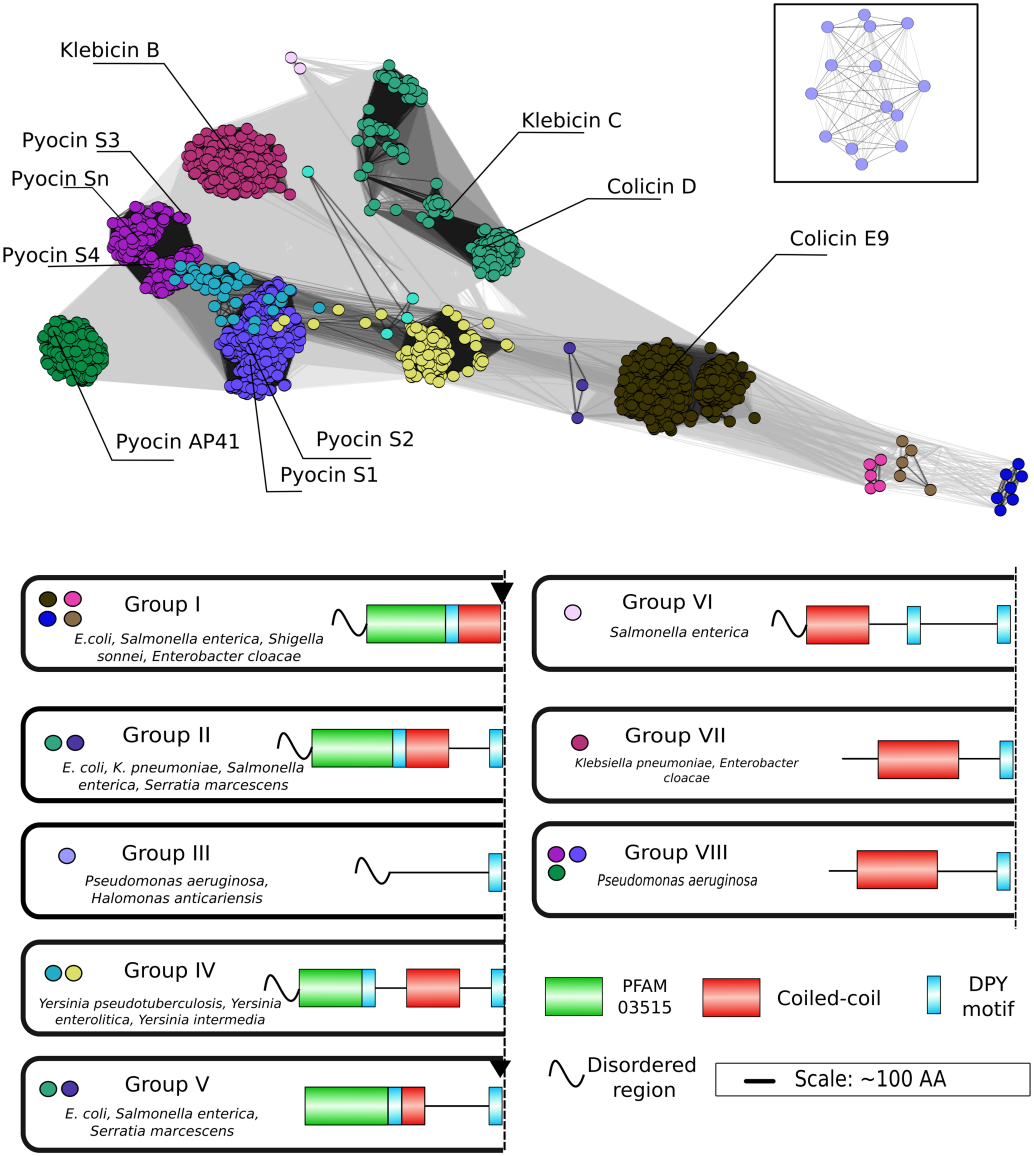
NBs cluster into 8 groups of differing domain arrangements. *Top panel*, sequences of NBs from *Enterobacteriaceae* and *Pseudomonas aeruginosa* had cytotoxic domains removed before clustering using CLANS [51]. *Bottom panel*, PFAM profiles, coiled-coil regions, disordered regions and the conserved DPY motif in the most common domain organisations observed for each species. Sequences within clades tend to share a similar predicted domain structure. Block arrows indicate proteolytic processing sites that have been identified for a select few NBs [52, 53]. Box inset contains pyocin S9 sequences which were too distantly removed to show at the correct distance. Connections show High Scoring Segment Pairs (HSP) of <5x10^-20^. The figure also highlights previously identified NBs as reference points. In addition to identifying many more NBs within the groups containing such characterized NBs we also identify three NB groups (III, IV and VI) that have completely novel domain organisations not previously described in the literature.

No systematic *in silico* analysis has been conducted to ascertain the diversity of bacteriocin protein sequences in bacteria. Domain organization in NBs has conventionally been based on that found in colicins and pyocins. Colicins are composed of an amino terminal T-domain, a central R-domain and a C-terminal cytotoxic domain (T-R-C), while pyocins are thought to have their T- and R- domains switched (R-T-C). Rather than assign functionality to domains, which is largely unknown in our dataset, we analyzed the NB protein sequences identified through our bioinformatics pipeline for structural elements/motifs commonly associated with NBs, including intrinsically disordered and coiled-coil regions and structured domains described in the PFAM database (Fig 3). For this analysis, the cytotoxic nucleases of the sequences, which are always at the C-termini of NBs, were excluded as they provide little information on domains involved in import. Our approach captured a large proportion of previously identified NBs reported in the literature thereby validating the pipeline. Our analysis revealed five principles that underpin the diversity of NB sequences involved in their import. First, with the exception of some *Pseudomonas* species, (which appear too diverse for meaningful analysis by CLANS therefore we present a phylogenetic analysis in S8 Fig) (see below), the majority of NB sequences (2866: ~93%) can be classified into eight groups (I-VIII) based on sequence identity and predicted domain organization. Second, six of the eight groups come from the *Enterobacteriaceae* (groups I, II, IV, V, VI and VII) and two from *P*. *aeruginosa* (groups III and VIII). Third, groups are generally not comprised of sequences from a single genus (e.g. group V includes *E*. *coli*, *Serratia marescens*, *Salmonella enterica* and *Klebsiella pneumoniae*) but can be populated entirely by a single genus (e.g. group VI contains only *Salmonella enterica* and group IV contains only *Yersinia* spp NBs). Conversely, some species appear in multiple groups (e.g. klebicins of *K*. *pneumoniae* are found in groups V and VII). Fourth, the degree of sequence identity within groups is high (40–90%) but is very low between groups (<20%). Fifth, only one sequence motif was common to all NBs across the eight groups, identified as the DPY motif in Fig 3 (see below). Four of the eight groups have duplicated DPY motifs (groups II, IV, V and VI) although the biological relevance of this is currently unknown. Below, we highlight some of the main structural features of NBs for a select few species that encode them.

*E*. *coli* NBs of the rRNase and DNase type were identified in the 1970s [54, 55] and are commonly referred to as E-type colicins [16]. The present analysis places these NBs in group I, which also includes NBs from *Klebsiella pneumoniae*, *Serratia marcescens*, *Shigella sonnei*, *Enterobacter cloacae*, *Xenorhabdus*, and *Citrobacter* (Fig 3). Structures for both the rRNase NB ColE3 and DNase NB ColE9 from this group have been reported [56], plus there have been numerous biophysical and structural studies of other NBs from this group [57]. The main structural features of group I NBs are a T-domain composed of a disordered N-terminal region adjoining a folded domain, the latter identified in the PFAM database as PFAM 03515, and a central coiled-coil region which constitutes the R-domain. In E-type colicins the R-domain binds the vitamin B_12_ transporter BtuB, while disordered regions (known as the Intrinsically Unstructured Translocation Domain or IUTD) are involved in binding OmpF in the outer membrane and TolB in the periplasm as part of their import mechanism [58, 59]. Group I NBs (apart from those within *Morganella*) have disordered regions at their N-terminus, which is also seen in five of the eight NB groups (Fig 3). It is likely these disordered regions are involved in the translocation mechanism of all NBs that contain them. Once in the periplasm the nuclease domains of NBs are translocated across the cytoplasmic membrane by the AAA+ ATPase/protease FtsH, which also proteolytically releases the nuclease to the cytosol [60, 61]. The processing site identified by de Zamaroczy and co-workers [52] can be found in the NBs of groups I and V.

*K*. *pneumoniae* is an important human pathogen and a leading cause of drug resistant infections. Multiple NBs, including four well characterized klebicins (A-D), have been identified in *K*. *pneumoniae* isolates [62]. We identified klebicins A (equivalent to cloacin DF13, group I), klebicin B (group VII) and klebicin C (group II), as well as two novel DNases which share less than 50% similarity to klebicin B or C at the N-terminus (group VII). Sequences within group VII contained a large coiled-coil region at the N-terminus whereas group II klebicins have much shorter coiled-coil regions similar to group V NBs, which includes the well-studied colicin D [29, 63]. Group II and group V NBs only differ in the presence of N-terminal disorder and overlap in terms of their sequence similarity clustering. The klebicins of group II contain two copies of the DPY motif whereas group VII NBs only have one.

*Serratia marcescens* is a ubiquitous environmental organism that has become a major cause of healthcare associated infections, with increasing reports of antimicrobial resistance [64, 65]. Only one *Serratia* bacteriocin (the pore forming marcescin 28b) has been identified to-date [66]. Using our pipeline, 11 NB families with no significant similarity to 28b were identified in 44 *Serratia* isolates. Bacteriocins containing either DNase and tRNase cytotoxic domains appeared to be interchangeable suggesting recombination of cytotoxic domains, a trait that is common in polymorphic toxins [67]. 8 of the 11 clusters were predicted to be chromosomally encoded and are present in group V. Three of the NBs are more closely related to cloacin DF13, containing an identifiable N-terminal T-domain and 42% similarity to the T- and R- domains of cloacin DF13 (group I), but have a long linker region and a repeat of the DPY motif and therefore form a separate group (group II).

The genus *Yersinia* has previously been shown to contain a number of bactericidal toxins only two of which have been characterized as bacteriocins. Pesticins from *Yersinia pestis* degrade peptidoglycan using murimidase activity and colicin F_Y_ is a pore forming bacteriocin [68, 69]. We identified 18 NB sequence clusters at 90% sequence identity in 8 *Yersinia* species and identified both DNase and rRNase cytotoxic motifs. None of these sequences had significant similarity to pesticin or colicin F_Y_. *Yersinia* NBs formed a separate group within the *Enterobacteriaceae*, the group IV NBs, although somewhat unexpectedly this did not include *Y*. *pestis*. These sequences have a ~90 residue unstructured region at the N-terminus similar to the IUTD of colicins and a long coiled-coil region, likely to be the receptor binding domain. 15 *Yersinia* NBs had a conserved region within the disordered N-terminal domain suggesting they may target the same translocation machinery.

*Pseudomonas aeruginosa* is an opportunistic pathogen and a leading cause of nosocomial infections [70, 71]. The S-type pyocins of *Pseudomonas aeruginosa* share a similar domain structure to colicins but with a rearrangement of their T- and R- domains and a small domain of unknown function between them [42]. An *in silico* investigation into the diversity of *Pseudomonas* toxins has previously identified several novel S-type pyocins [44]. Similar to this earlier study, we identified pyocins S1-9 (excluding the pore former pyocin S5) and S11-12, as well as two additional recombinations; pyocin S13 (or SD1) which contains the colicin D like cytotoxic domain of S11 and S12 associated with the N-terminus of S1 [72], and a pyocin we term S3C which contains the rRNase domain of colicin E3 associated with the pyocin S3 N-terminal domains and has not been reported previously in literature (Fig 3). Two novel HNH DNase motifs were also identified with 40–60% similarity to the S1, S2 and AP41 cytotoxic domains. Overall, 85% (874/1024 genomes) *P*. *aeruginosa* strains contained NB genes, which largely segregate to a single group in our analysis (group VIII). A second, much smaller, group (group III) is populated only by a single pyocin (S9), also identified by Ghequire et al [44]. These proteins are predicted to have a disordered N-terminal domain, a single DPY motif and no identifiable coiled-coil region.

NBs were identified in 20 *Pseudomonas* species other than *P*. *aeruginosa*. NBs containing an rRNase domain were identified in *P*. *fluorescens*, *P*. *synxantha*, *P*. *poae*, *P*. *brassicacearum* and 10 *Pseudomonas* sp. isolates. In total, 175 *Pseudomonas* spp. (excluding *P*. *aeruginosa*) genomes contain a DNase or E3-type rRNase domain (51.5% and 21%, respectively). NBs with a tRNase cytotoxic domain were not identified outside of *P*. *aeruginosa* species which is in line with previous analysis [44] (S8 Fig). Many of the species observed were represented in the database by only a single genome suggesting the true extent of the sequence diversity within *Pseudomonas* spp. has yet to be established. Ghequire *et al* [44] also reported the presence of a group of NBs that contain a dual tandem repeat of the so-called pyocin_s domain (see below), followed by a DNase domain (either HNH or non-HNH) in 10 isolates. We observed a similar domain organisation but note that the additional DNase domain does not contain a functional HNH motif.

### The DPY motif—A conserved epitope implicated in NB translocation to the cytoplasm

**Fig 4:**
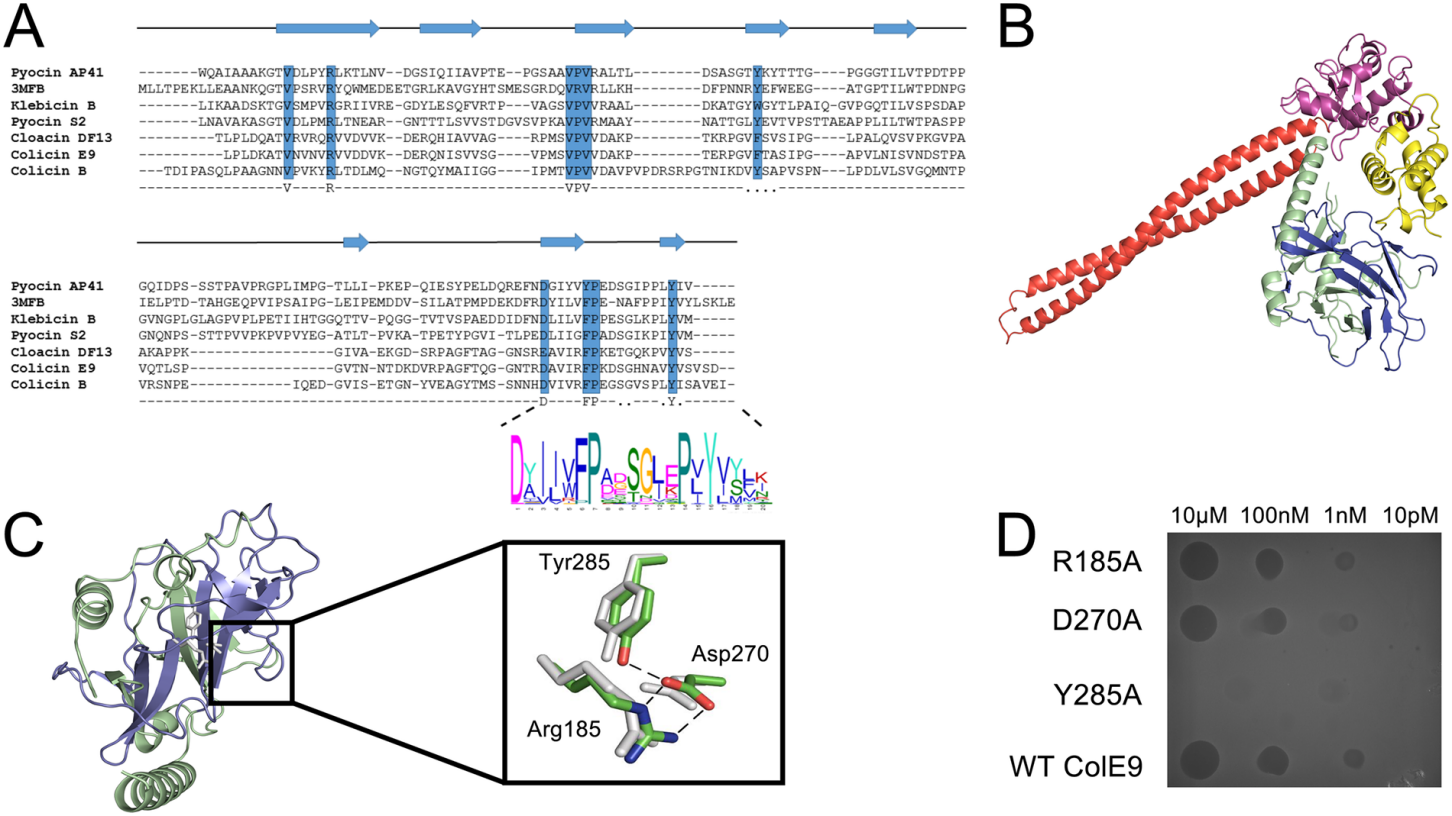
A conserved translocation motif was identified in all NBs. ***a***, Protein structure-based sequence alignment using PROMALS 3D indicates the conserved β-sheet secondary structure of a conserved domain identified in nuclease bacteriocins. Alignment features bacteriocins from *E*. *coli* (Colicin B, E9 and Cloacin DF13); *Klebsiella pneumoniae* (klebicin B), *Pseudomonas aeruginosa* (Pyocin AP41) and the pyocin_s domain from *Erwinia carotovora* The DPY motif was identified using MEME and is shown by a LOGO plot at the C-terminal end of the T-domain. ***b***, Crystal structure of colicin E9, with its constituent domains identified, in complex with its immunity protein Im9 (PDB code, 5EW5). ***c***, The conserved segment of the T-domain (*blue*) is annotated as the pyocin_S domain in the PFAM database (PFAM 06958), which is usually part of a larger T-domain, annotated as PFAM 03515 (*green*). *Inset*, Alignment of resides at the core of PFAM 06958 showing a conserved hydrogen bond network formed between the residues of the DPY motif; Asp270 and Tyr285 (colicin E9 numbering) and Arg185 at the beginning of PFAM 06958. ***d***, Cytotoxic plate killing assay of DPY motif mutations. 100-fold serial dilutions of colicin E9 and DPY motif alanine mutants were spotted onto a lawn of sensitive *E*. *coli* showing that only the Tyr285Ala mutant abolishes colicin activity.

We analyzed the 3094 NB sequences captured by our bioinformatics pipeline using MEME [73] and identified a ~15-16-residue motif (D-X_4_-FP-X_8_-Y) located within the T-domain of nuclease colicins, which we refer to as the DPY motif (Fig 4a). T-domains of NBs such as colicins are typically ~300 amino acids in length and share very low sequence identity (<15%), with many loop insertions/deletions between the β-sheet secondary structure elements. The DPY motif is found in all NB sequences we examined and as such is a clear identifier of T-domains, the first of its kind. Sano *et al*. [20] previously highlighted a similar region in a few nuclease pyocins and colicins the deletion of which abolished cytotoxic activity [74]. The structures of three NB T-domains are known (annotated in the PFAM database as PFAM 03515); colicin E3 (2B5U), colicin E9 (5EW5 [56]) and the S-type pyocin domain from *Erwinia carotovora* (3MFB) as well as the T-domain of the pore forming colicin B (1RH1) which shares high sequence identity to the T-domain of the NB colicin D. We performed structure-based sequence alignments and found that the DPY motif is integral to a much larger segment spanning the C-terminal half (~140 amino acids) of the T-domain (Fig 4b). This segment (coloured blue in Fig 4b) is identified in the PFAM database as the pyocin_s domain (PFAM 06958) [42]. Nine amino acids identified within PFAM 06958 are conserved across the T-domains of all NBs, which are coincident with five β-strands of the structure (Fig 4a). Finally, it is interesting to note that the location and indeed number of DPY motifs within NBs varies; it is generally placed at the C-terminal end of T-domains, which can be proximal or distal to the cytotoxic domain of NBs (Fig 3).

The DPY motif forms a buried hydrogen bond network within PFAM 06958 that in colicin E9 involves Tyr285 and Asp270, which is salt-bridged to Arg185 at the N-terminal end of PFAM 06958 (Fig 4c). We mutated these three residues to alanine separately and in combination and found that mutation of Tyr285 (by itself or in combination) abolished cell killing whereas Asp270 and Arg185 mutations had no observable effect (Fig 4d).

With the exception of colicin B, a pore-forming colicin that shares a common ancestry with the nuclease colicin D, the DPY motif is found exclusively in NBs. The motif is not present in bacteriocins that are active in the periplasm such as the pore-forming colicins E1, A, Ia, Ib and pyocin S5 and lipid II hydrolases such as colicin M. Hence, the DPY motif is specific to bacteriocins active in the cytoplasm. The involvement of the motif in translocation to the cytoplasm could be either at the outer membrane step, defining a path through the cell envelope, or the inner membrane, in a step prior to FtsH-mediated translocation of the nuclease to the cytoplasm.

### Testing the activity of a novel nuclease bacteriocin

The ability of the pipeline to predict novel bacteriocins could improve our repertoire of potential species-specific antimicrobials for a number of clinically relevant species. As a test of the pipeline, we cloned and expressed a novel bacteriocin from *Pseudomonas aeruginosa*, which we call pyocin Sn. Pyocin Sn (832 residues) resides within group VIII, has no detectable disorder domain, is characterized by a large coiled-coil region and a C-terminal, non-HNH type DNase domain. Purified pyocin Sn had cytotoxic activity against three different strains of *P*. *aeruginosa* (S9 Fig). Hence our pipeline accurately identifies novel NBs in bacterial genomes.

### Association of NBs with virulence factors in *Escherichia coli*

**Fig 5:**
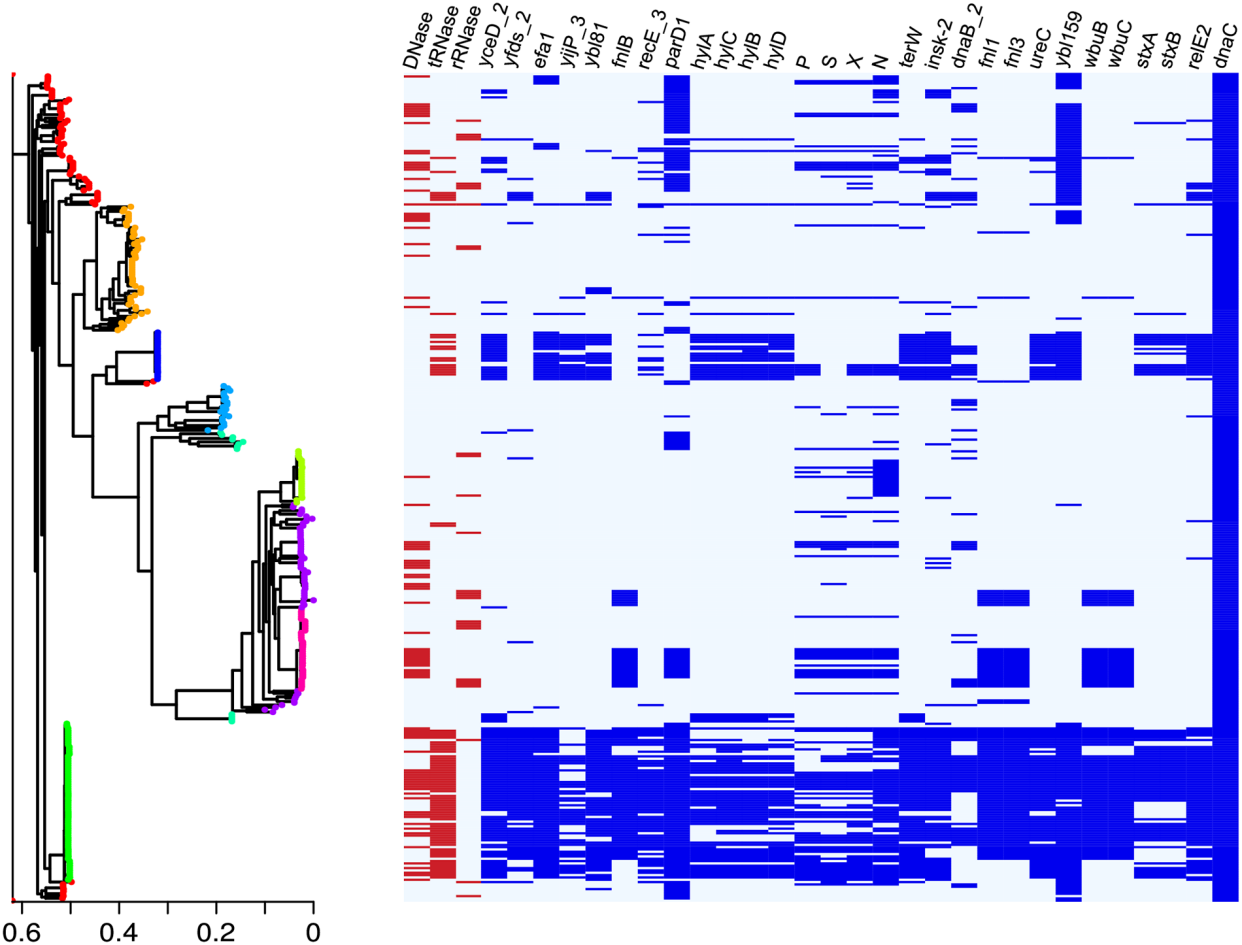
Pangenome analysis of colicinogenic bacteria shows evidence of an association between NBs and virulence factors. Association of pathogenicity and colicinogenicity genes based on a Cochran-Mantel-haenszel test. *Left-hand panel*, RAxML tree of a core genome alignment. Population structure was calculated using BAPS and tree nodes are coloured by cluster as predicted by BAPS. *Right-hand panel*, presence and absence of genes associated with colicinogenicity. Red genes show nuclease colicin of different cytotoxic domains. dnaC is a core gene and included as a control.

Recent work has highlighted the potential role of colicins as virulence factors in pathogenic bacteria, which can be used to displace commensal bacteria [24, 25]. As yet however there has been no assessment of whether specific bacteriocins such as colicins are associated with pathogenicity. To look for an association between colicinogenicity and virulence we calculated the pangenome of 357 *E*. *coli* strains (166 colicinogenic strains and 191 non-colicinogenic strains) using the roary pangenome. 41,723 gene groups were identified (1905 core genes (100–99%), 739 soft core genes (99–95%), 3,500 shell genes (95–15%) and 35,579 cloud genes (15–0%)). Association was determined by a fisher’s test followed by a Cochran-Mantel-Haenszel on BAPS clustered populations to correct for population stratification. 28 genes (excluding genes that code for a colicin, immunity protein or plasmid mobility genes) were found to be significantly associated with the trait of colicinogenicity (Table 1). These genes included virulence factors, toxin/anti-toxin modules, phages and genes involved in LPS and O-antigen biogenesis (Fig 5). Specific virulence factors are associated with pathotypes of colicinogenic *E*. *coli* such as the shigella toxin producing *E*. *coli* STEC and the enterohemorrhagic *E*. *coli* factor for adherence. Other genes associated with the carriage of colicin genes are general virulence factors such as hemolysins, which target the eukaryotic plasma membrane, and ureases that increase cytoplasmic pH to allow *E*. *coli* to survive in acidic environments. We also identify an association with phage genes suggesting either horizontal transfer of NB genes via bacteriophages or that associated phage lysis genes are being recruited for release of NBs, as has been proposed for other NBs [33]. The association of NBs with virulence factors suggests a role in the pathogenicity mechanisms of *E*. *coli*. This *in silico* evidence supports the hypothesis that bacteria use bacteriocins to displace native microflora in order to colonize and cause infection.

**Table 1:** Gene clusters identified by Roary pangenome software and shown as significantly associated with nuclease colicins.

Cluster	Description
***Virulence factors***	
*yijP_3*	Uncharacterized protein. Shown to be involved in invasion of brain endothelial cells [75]
*stxA/B*	Shigella toxin/antitoxin
*terW/E*	Tellurium resistance protein
*ureC*	Urease
*hyl A/B/C/D*	Hemolysin operon
*efa1*	*Escherichia coli* factor for adherence
***O-antigen biosynthesis***	
*fnlB*	UDP-l-FucNAc biosynthesis pathway [76]
*fnl1*	UDP-l-FucNAc biosynthesis pathway
*fnl3*	UDP-l-FucNAc biosynthesis pathway
*wbuB*	l-FucNAc transferase
***Phage***	
P	Phage terminase
X	Phage tail
S	Phage tail

In summary, we have developed and validated a pipeline for the identification of NB genes in bacterial genomes. Using this pipeline, we are able to show that the five NB families are widespread in γ-proteobacteria and that bacteria containing these genes occupy diverse ecological niches. Most have yet to be characterized. We also show that while NBs have high sequence diversity and multiple domain arrangements, they retain a conserved motif that is likely required for translocation of the nuclease to the cytoplasm. Finally, using a pangenome analysis of *E*. *coli* isolates we show that NB genes are associated with virulence factors supporting the hypothesis that NBs are exploited by invasive bacteria to displace host microflora.

## Materials and methods

### Genomic databases

Nucleotide fasta files for genomes from PATRIC [40] and NCBI assembled bacteria are freely available from ftp.patricbrc.org/patric2 (accessed 11/07/2014) and ftp.ncbi.nlm.nih.gov/genomes/ASSEMBLY_BACTERIA (accessed 14/05/2015), respectively. We also made use of the PubMLST Multispecies isolate database website (https://pubmlst.org/rmlst/ accessed 10/12/2014) [77]. All databases were subjected to the nuclease bacteriocin pipeline however due to the size and coverage of the pubMLST database (10-fold larger then PATRIC) results are only shown for pubMLST. PATRIC was used to calculate environmental association as it has greater metadata and NCBI used to identify Ton/Tol operons as genomes are assembled. PATRIC and NCBI databases did not produce any novel NBs that were not identified in pubMLST. The PubMLST Multispecies isolate database contains NCBI Nucleotide database (finished genomes) and community contributed NCBI assembled genomes (NCBI Assembly database) as well as genomes assembled in-house from the publically available raw read data at the European Nucleotide Archive (ENA-SRA). The PubMLST Multispecies isolate database aims to collect a wide range of bacterial species from all bacterial Classes that have been NGS sequenced in order to reflect the current knowledge of bacterial genomes and does not explicitly bias itself towards any particular subset of bacteria; however, it is subject to any bias of sequences deposited in the ENA. PubMLST Multispecies isolate database genomes are assembled in-house from the ENA-SRA database. The short read sequences were assembled using the Velvet genome assembly program (v1.2.08) [78]. All odd-numbered kmer lengths between 21 and the read length were sampled using the VelvetOptimiser software (v2.2.4, bioinformatics.net.au/software.velvetoptimiser.shtml) to automatically calculate the optimal assembly parameters for Velvet. Default parameters were used throughout with the exception that no scaffolding was performed and only contigs with 200bp or more were included in the final assembly. Data from pubMLST used in this study including source (NCBI or ENA-SRA) and accession within source database is provided (S1 Data).

### Developing a pipeline to detect nuclease NBs and associated immunity proteins

Profile pairs shown in S1 Table were used to search for homologues of a NB and immunity protein in each of the translations using HMMscan (HMMer3.1) with default search parameters (http://hmmer.org/). PFAM profiles were used for all five cytotoxic domains, HNH DNase, the non-HNH DNase pyocin S3 cytotoxic domain, tRNase (both Colicin D-like and Colicin E5-like) and rRNase [79]. To test for sensitivity, NBs identified in a first iteration of the pipeline were used to make secondary profile pairs (iterated HMM). Regions that matched the conserved motifs were extracted using the easel toolkit associated with HMMer3.1. Sequence alignments were performed using either MUSCLE [80] or CLUSTAL Omega [81].

Genomes from all databases were submitted through the pipeline as shown in S3 Fig. Briefly, genomes were translated into six frames. Each frame was aligned to the profile pairs in S1 Table using HMMer3.1 HMMscan with default parameters E-value cut off 5x10^-2^. Custom python scripts were used to find the intergenic distance, here defined as the bases between the end of the cytotoxic profile alignment and the start of the immunity profile alignment. We do not measure the actual intergenic region between two open reading frames, as ORFs for immunity genes are often not identified by annotation software. For all cytotoxic domains apart from the ColE5 tRNase profile pairs with intergenic distances of greater than 60 base pairs were discarded. For the ColE5 like tRNase cytotoxic domain the distance was extended to 200bp as the profiles did not cover the end of the cytotoxic domain and the beginning of the immunity leading to an extended region between the two (S2 Fig). Open reading frames containing the cytotoxic and immunity pairs were extracted using Prodigal v2.6.1. ORFs greater than 950 residues and less than 350 residues were discarded as non-NB ORFs [13]. Sequences were aligned to the PFAM-A version 27 profile database. ORFs with significant alignments (E-value <5x10^-5^) to active secretion systems or profiles not biologically relevant to NBs were rejected. To correct for incorrectly predicted ORFs we cluster sequences using cd-hit to find the longest ORF prediction for each sequence. Two additional profiles were used to discriminate T6SS from *Vibrio* and *Klebsiella*. A profile built containing the recently described MIX domain of Vibrio T6SS. A second profile was generated to remove T6SS effectors from *Klebsiella pneumoniae* which were located downstream of a T6SS Rhs protein.

### Determining chromosomal or plasmid association of NB genes

Conserved replicon sequences from the Carattoli typing scheme were downloaded from Plasmid Finder [45]. In addition, a conserved region from the ColE1 replicon were added [45]. An E-value cut off of 5x10^-5^ was used. Sequenced plasmids from NCBI and EBI were downloaded to generate a custom blast database of 3603 plasmids. Contigs containing a suspected NB were aligned to the database using blastn with default settings. Regions of the contig that aligned to a plasmid (E value < 5x10^-7^) were summed and overlaps removed to determine the percentage of plasmid association. A small number of contigs could be verified using the NCBI fully assembled genomes that contain fully assembled chromosomes and plasmids. To detect horizontal gene transfer by colicinogenic plasmids found across multiple genomes, sequences were clustered using cd-hit with sequence identity of 90% and genome comparisons were performed using MAUVE2 [82].

### Identifying Ton and Tol operons in a diverse set of bacterial genomes

2785 fully assembled bacterial genomes with open reading frames predicted using Glimmer3.0 were downloaded from NCBI. S3 Table describes the PFAM profiles (Pfam 27) used to identify conserved regions within proteins of the Ton/Tol operons. HMMscan (HMMer3.1) was again used to identify profile matches in the open reading frames (E-value cutoff 5x10^-5^). Within each genome, regions of interest were identified by clustering co-ordinates of profile matches along the chromosome with a threshold of 2000 bases forming a new cluster. To differentiate between Ton, Tol and Mot operons, simple presence/absence rules were applied. For a region of interest to be classified as a Tol operon it needed to contain ExbD, MotA_ExbB and PD40. Ton operons were defined as containing ExbD, MotA_ExbB, TonB_C but not the PD40 repeat which is a structural motif within TolB that does not have a homologue in the Ton operon.

### Clustering NB proteins by CLANS analysis

Cytotoxic domains were removed from bacteriocin sequences by removing the section of sequence that overlapped with the Pfam cytotoxic domains using HMMsearch and the easl toolkit. Sequences with cytotoxic domains removed were clustered using CLANS. Colored clusters were calculated using network approach where each sequence emits the clusters it is linked to weighted by the negative log of the p-value. This is iterated until clusters experience no further change. Only HSP hits p<1x10^-10^ were used in the analysis [51]. Coiled-coil regions were predicted using COILS [83]. Disorder was predicted using IUPRED [84]. Disordered regions were defined as predictions of long disorder greater than 0.5 stretching for ≥ 30 residues.

### Pangenome analysis

357 *E*. *coli* genomes were accessed from the ENA-SRA (S2 Data) and assembled using a Velvet based in—house assembly and improvement pipeline before annotation using Prokka [85]. Pangenome analysis was performed using the Roary pangenome software [86] and with similarity cut-off set at 95%. Phylogenetic trees were calculated from the core gene alignment produced by Roary using RAxML (WAG model with Gamma correction) [87]. The R stats package was used to perform the analysis. Gene clusters were tested for association with colicinogenic genomes by a fisher’s exact test. Population stratification was evaluated using BAPS [88] clustering and a Cochran-Mantel-haenzsel test.

### Prediction of PFAM domains

PFAM (Pfam 27) domains from were predicted using HMMscan (HMMer3.1).

### Cytotoxic plate killing assay

Killing assays were performed by inoculating soft LB agar with either colicin sensitive *E*. *coli* JM83 for DPY mutations or one of three *P*. *aeruginosa* strains (PAO1, UCBPP-PA14 or PA14) for pyocin Sn sensitivity, and overlaid on LB agar. Serial dilutions were prepared using 20mM Tris-HCl pH 7 and 2 μl spotted onto the agar. Plates were incubated overnight at 37°C and scored on presence of a zone of clearance in the soft LB agar.

### Cloning and expression of the novel bacteriocin Pyocin Sn

Codon optimised synthetic genes encoding pyocin Sn (GenBank: AHA26272.1) and its immunity protein in series (Eurofins Genomics) were ligated into the NdeI / XhoI sites of pET24a (Novagen) to give pNGH252 such that the immunity protein contained a C-terminal hexa-histidine tag. A second copy of the immunity protein, again with a C-terminal hexa-histidine tag, was ligated into the NcoI / HindIII sites of pACYCDuet-1 (Novagen), to give pNGH260. Both plasmids were transformed into BL21 (DE3) cells to ensure the immunity protein expressed in excess of the bacteriocin. Cultures of pNGH252 pNGH260 BL21 (DE3) were grown at 28°C in LB 50 μg/ml kanamycin, 34 μg/ml chloramphenicol to an OD600 nm of 0.7 upon which expression from both plasmids was induced through the addition of IPTG to a final concentration of 1 mM. Cells were grown for a further 16 hours, before being harvested by centrifugation, and lysed through sonication in 25 mM Tris-HCl, pH 7.5, 500 mM NaCl, and 1 mM PMSF. Cell debris was removed through centrifugation at 17,500 xg with the supernatant being passed through a 0.45 μm filter. The pyocin Sn-Im_His6_ complex was purified on a 5 ml HisTrap FF column (GE Healthcare) equilibrated in 25 mM Tris-HCl, pH 7.5, 150 mM NaCl eluting bound protein with a 0 to 250 mM imidazole gradient over 10 column volumes. The pyocin Sn-Im_His6_ complex was further purified on a HiLoad 26/60 Superdex 200 column (GE Healthcare) equilibrated in 25 mM Tris-HCl, pH 7.5, 150 mM NaCl. The purified complex was quantified through A280 nm using a sequence based extinction coefficient of 70,250 M^-1^.cm^-1^.

## Supporting information

S1 Fig Cytotoxic domains targeted by the pipeline. Multiple sequence alignments of cytotoxic domains analysed by the pipeline showing the conserved motifs of both the toxin nuclease domain and immunity proteins identified the HMM profiles. In total, five profile pairs, three for RNases and two for DNases, were created that captured all known NB types. Fig 1 (main text) shows the profile pair for HNH DNases and their immunity proteins.
(TIF)

S2 Fig Distribution of intergenic regions between the cytotoxic HMM profiles and their associated HMM profiles of downstream immunity genes. Profile pairs with an intergenic region greater than 60bp (or 200bp for the case of ColE5 tRNases) were discounted from the analysis. a) Distribution for the HNH DNase and associated immunity gene. b) ColD tRNase, c) Non-HNH DNase, d) ColE5 tRNase, e) rRNase.
(TIF)

S3 Fig Pipeline for identifying and validating bacteriocin/immunity profile pairs. Numbers shown indicate the number of pairs accepted or rejected at each step for the pubMLST database using all 5 PFAM bacteriocin/immunity profile pairs.
(TIF)

S4 Fig The proportion of bacteria from each environment that were found to contain a NB. The PATRIC database contains metadata on the isolate environment of ~10% of bacteria. Environments are split to show contributions of the three most prevalent NB containing bacteria. (Sample sizes: Animal: 210, Blood: 527, Environmental: 641, GI/Stool: 1174, Human: 194, Lung: 385, Plant: 125, Soil: 116, Urine 778).
(TIF)

S5 Fig Association of contigs to plasmid sequences. A) NB containing contigs were compared to the Plasmid finder database. Bars indicate the number of contigs which contained both an NB gene and a conserved plasmid sequence. B) Association of NB containing contigs to the EBI/NCBI plasmid database. Each dot represents one contig.
(TIF)

S6 Fig Distribution of NBs is restricted to the γ-proteobacteria. Taxonomic tree representing all species in the pubMLST that have over 100 genomes, constructed using NCBI taxonomy commontree. *Red bar* indicates species that contained at least one NB.
(TIF)

S7 Fig Distribution of the Ton and Tol operons throughtout the bacterial kingdom. Taxonomic tree of assembled bacterial genomes from NCBI. Tree was constructed using NCBI commontree, blue bars indicate the presence of a Ton or Tol operon.
(TIF)

S8 Fig Phylogenetic analysis of NBs identified within *Pseudomonas* spp. Sequences from *Pseudomonas* spp. were aligned using MUSCLE and trees built using Mega 6.0 using the neighbour-joining algorithm with 1000 bootstrap replicates. Red boxes indicate the position of the pyocins of *P*. *aeruginosa*.
(TIF)

S9 Fig Cytotoxic activity of a novel pyocin, pyocin Sn, targeting *P*. *aeruginosa* identified through the pipeline. Pyocin Sn (Group VIII, Fig 3) is a non-HNH type DNase bacteriocin (88.8 kDa) newly identified by our bioinformatics analysis. As a test of the validity of this identification, *pyocin Sn* in combination with its immunity protein was overexpressed and purified from *E*. *coli* extracts and its activity against three strains of *P*. *aeruginosa* demonstrated. See Materials & methods for further details.
(TIF)

S1 Table HMM profiles used to identify the conserved motifs of NB cytotoxic domains and associated immunity proteins. PFAM profiles were first used to identify NBs. To test the robustness of our strategy, iterated profiles were created from the newly identified NBs and used in a second search.
(PDF)

S2 Table Distribution of NBs amongst species. Species that were identified as encoding a NB within their genome and the frequency of occurrence.
(PDF)

S3 Table HMM profiles used to identify the conserved regions of proteins in the Tol/Ton operons. Tol was differentiated from Ton by the presence of a PD40 repeat that is part of the TolB protein.
(PDF)

S1 Data PubMLST Multispecies database genomes used in this study. Over 100,000 genomes from multiple data source were collected and assembled in-house to form the database.
(XLSX)

S2 Data ENA accessions used in the pangenome analysis of *E*. *coli*. 357 *E*. *coli* accessions accessed from the ENA were used in the pangenome analysis.
(TXT)
