# Supplementary figures and images for: Diversity and distribution of nuclease bacteriocins in bacterial genomes revealed using Hidden Markov Models
Source: PLoS Comput Biol. 2017 Jul 17;13(7):e1005652. doi: 10.1371/journal.pcbi.1005652 (PMC5536347; doi:10.1371/journal.pcbi.1005652)

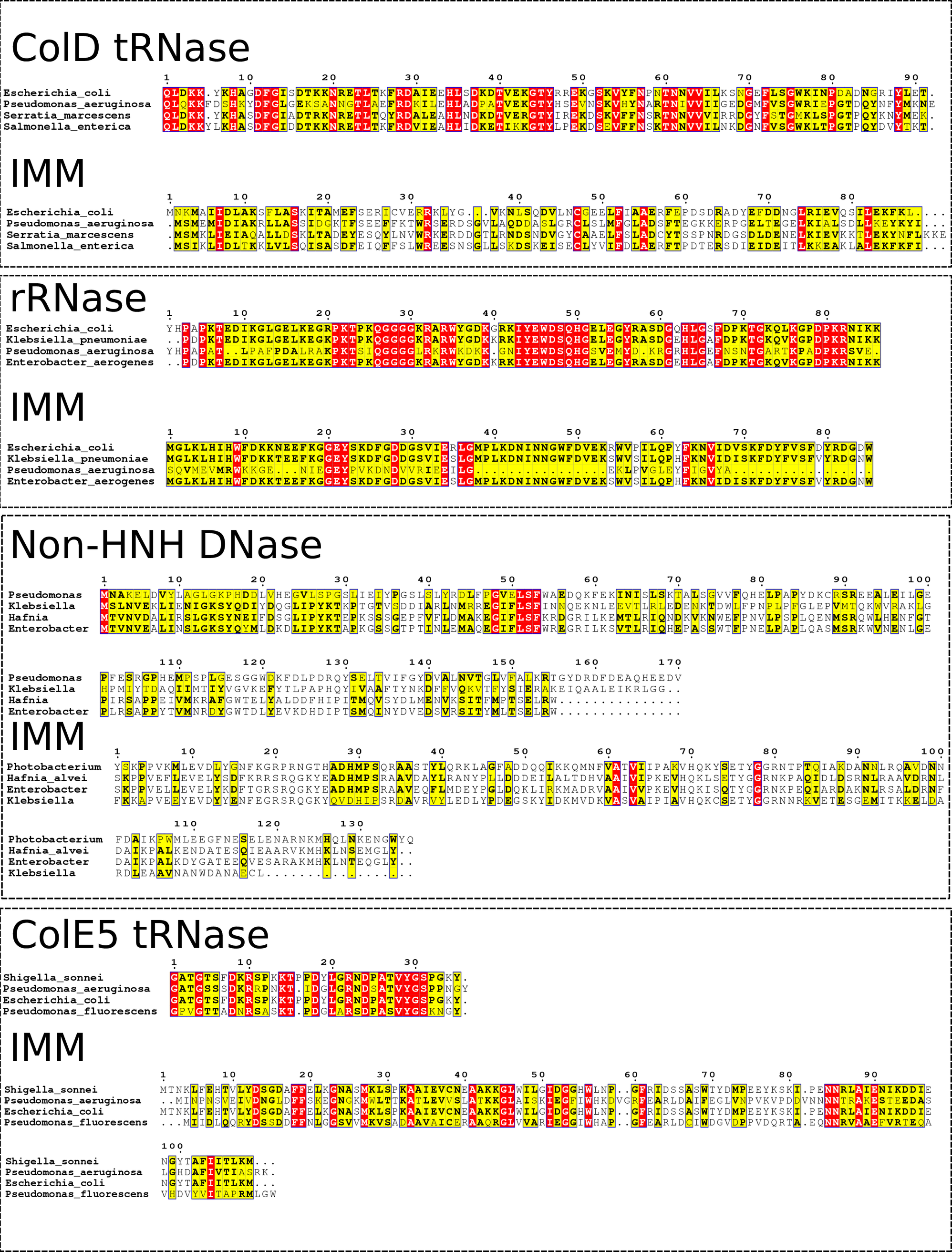

Supplement: S1 Fig — Multiple sequence alignments of cytotoxic domains analysed by the pipeline showing the conserved motifs of both the toxin nuclease domain and immunity proteins identified the HMM profiles. In total, five profile pairs, three for RNases and two for DNases, were created that captured all known NB types. Fig 1 (main text) shows the profile pair for HNH DNases and their immunity proteins. (TIF) [file pcbi.1005652.s001.tif]

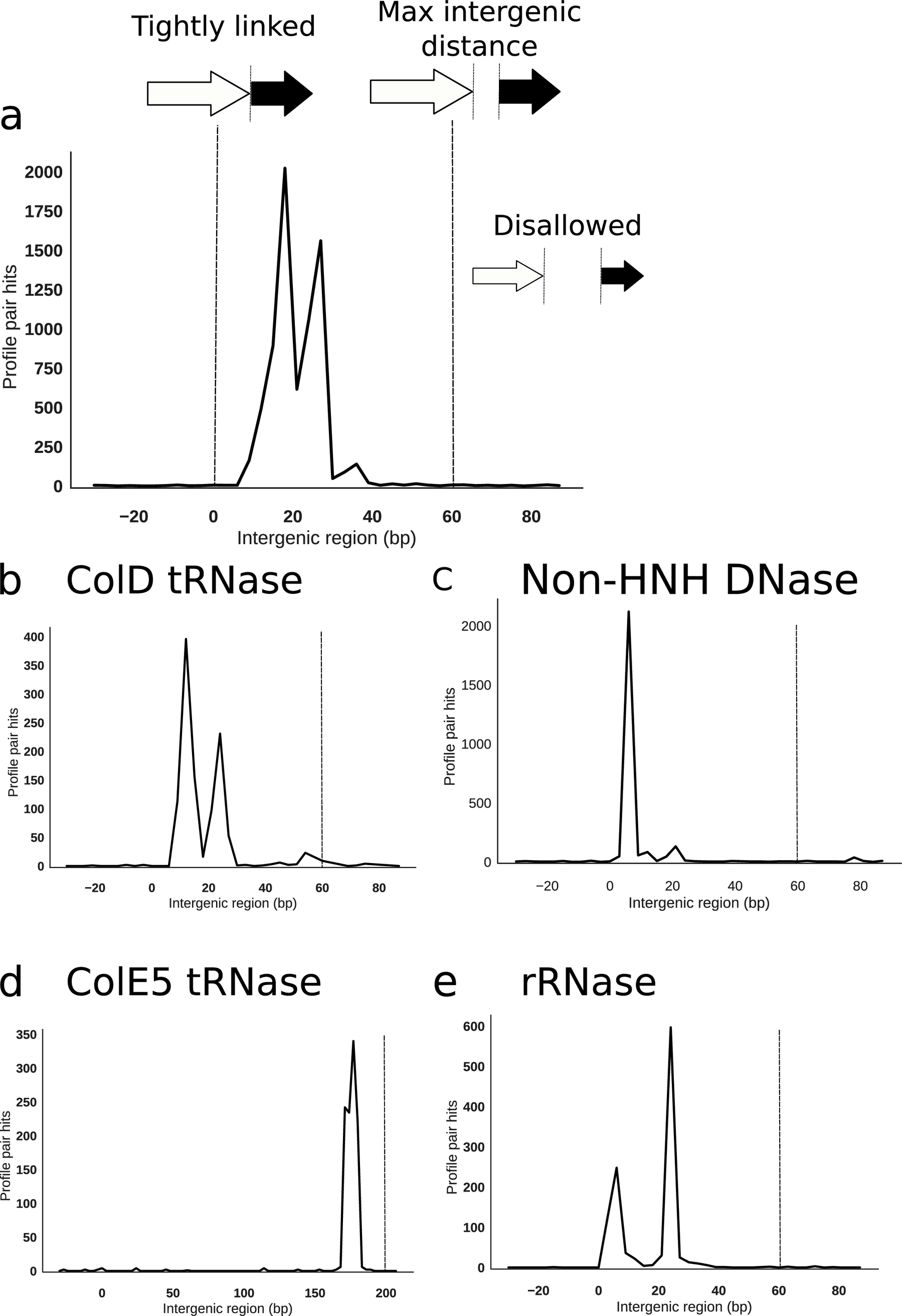

Supplement: S2 Fig — Profile pairs with an intergenic region greater than 60bp (or 200bp for the case of ColE5 tRNases) were discounted from the analysis. a) Distribution for the HNH DNase and associated immunity gene. b) ColD tRNase, c) Non-HNH DNase, d) ColE5 tRNase, e) rRNase. (TIF) [file pcbi.1005652.s002.tif]

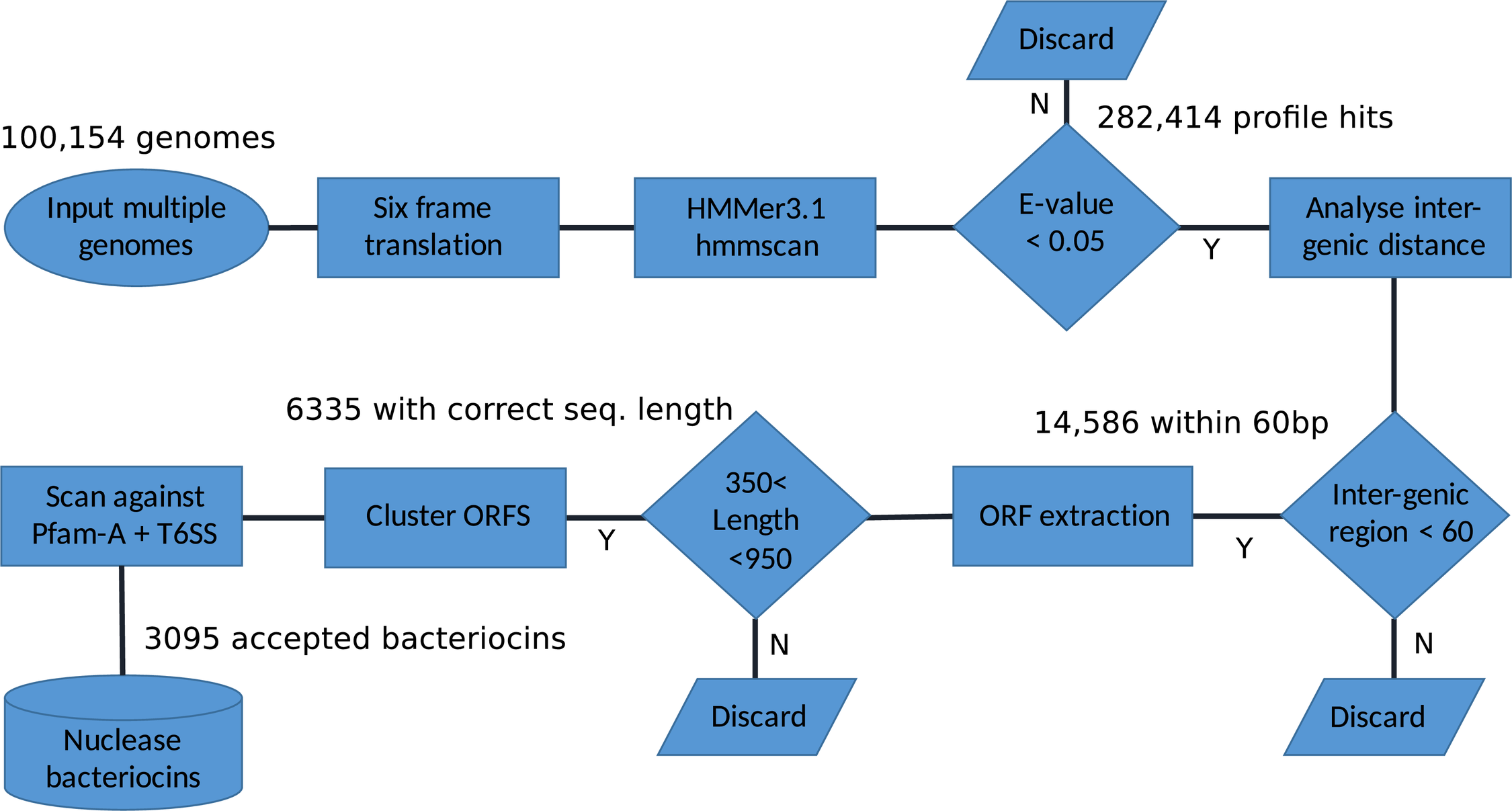

Supplement: S3 Fig — Numbers shown indicate the number of pairs accepted or rejected at each step for the pubMLST database using all 5 PFAM bacteriocin/immunity profile pairs. (TIF) [file pcbi.1005652.s003.tif]

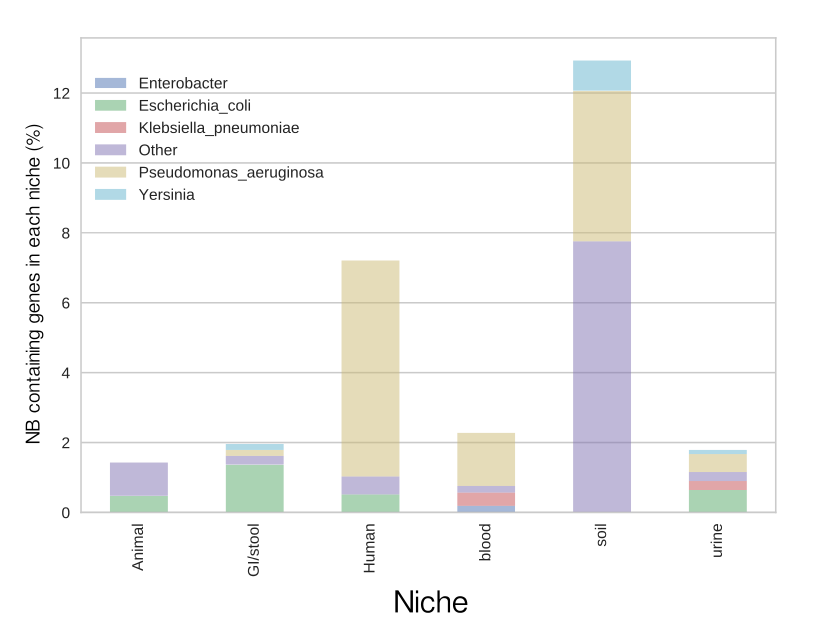

Supplement: S4 Fig — The PATRIC database contains metadata on the isolate environment of ~10% of bacteria. Environments are split to show contributions of the three most prevalent NB containing bacteria. (Sample sizes: Animal: 210, Blood: 527, Environmental: 641, GI/Stool: 1174, Human: 194, Lung: 385, Plant: 125, Soil: 116, Urine 778). (TIF) [file pcbi.1005652.s004.tif]

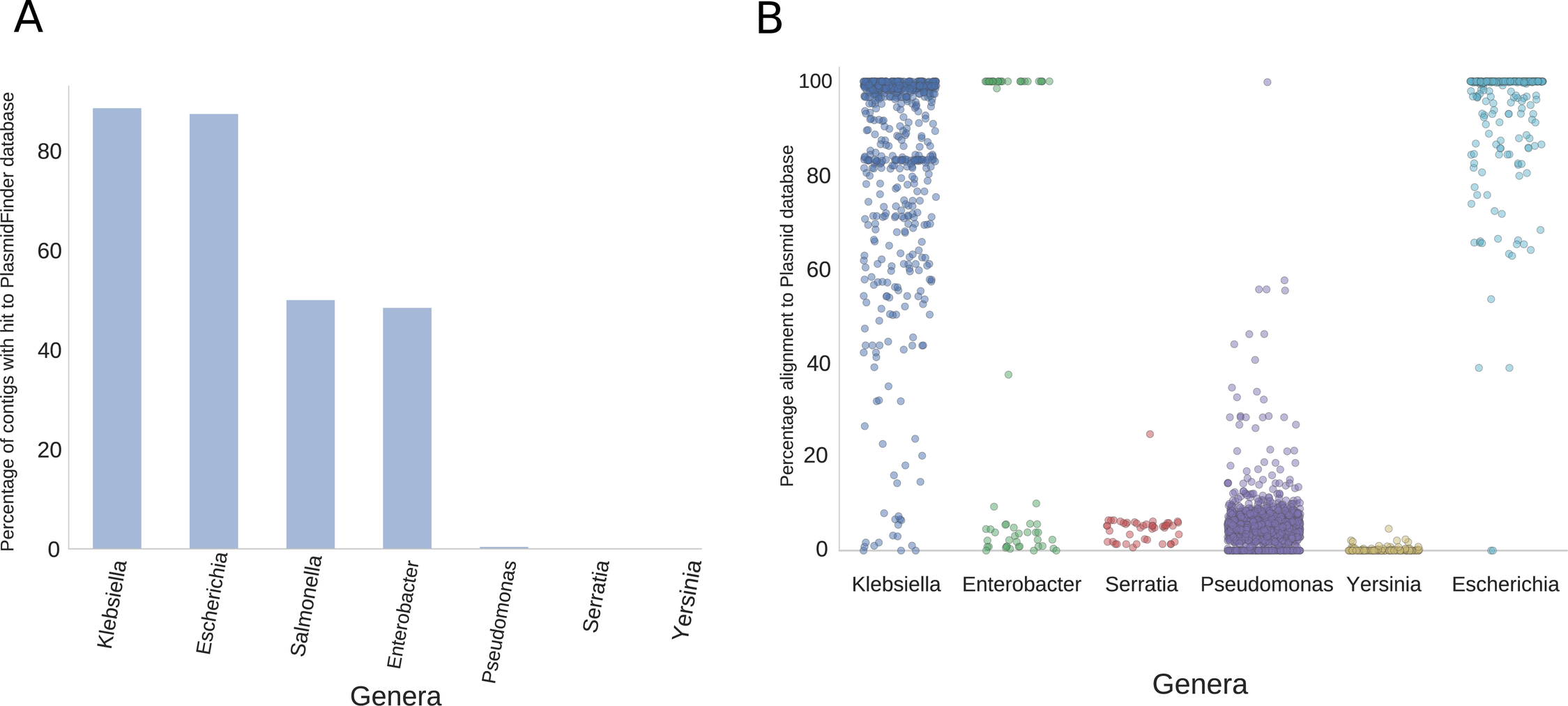

Supplement: S5 Fig — A) NB containing contigs were compared to the Plasmid finder database. Bars indicate the number of contigs which contained both an NB gene and a conserved plasmid sequence. B) Association of NB containing contigs to the EBI/NCBI plasmid database. Each dot represents one contig. (TIF) [file pcbi.1005652.s005.tif]

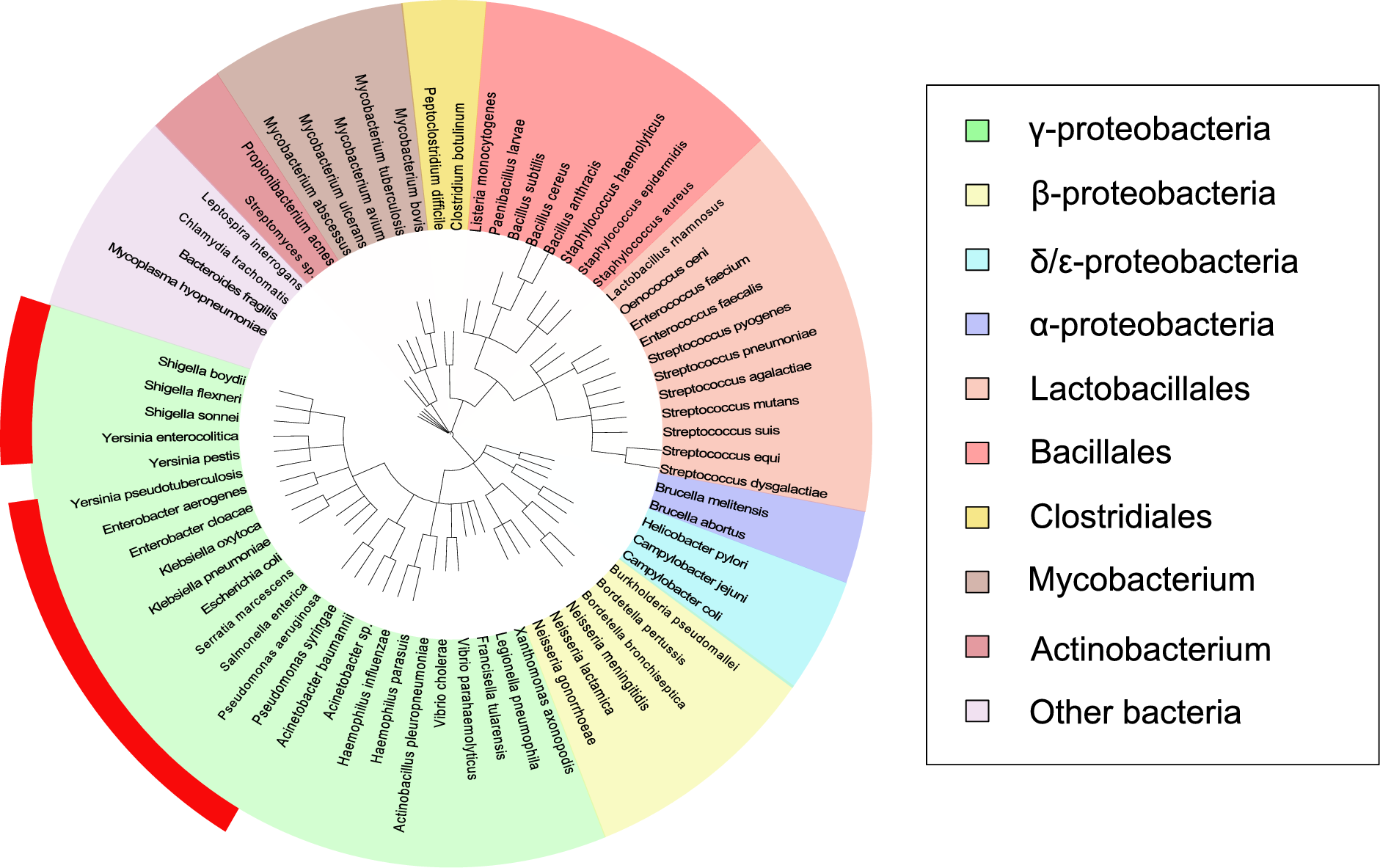

Supplement: S6 Fig — Taxonomic tree representing all species in the pubMLST that have over 100 genomes, constructed using NCBI taxonomy commontree. Red bar indicates species that contained at least one NB. (TIF) [file pcbi.1005652.s006.tif]

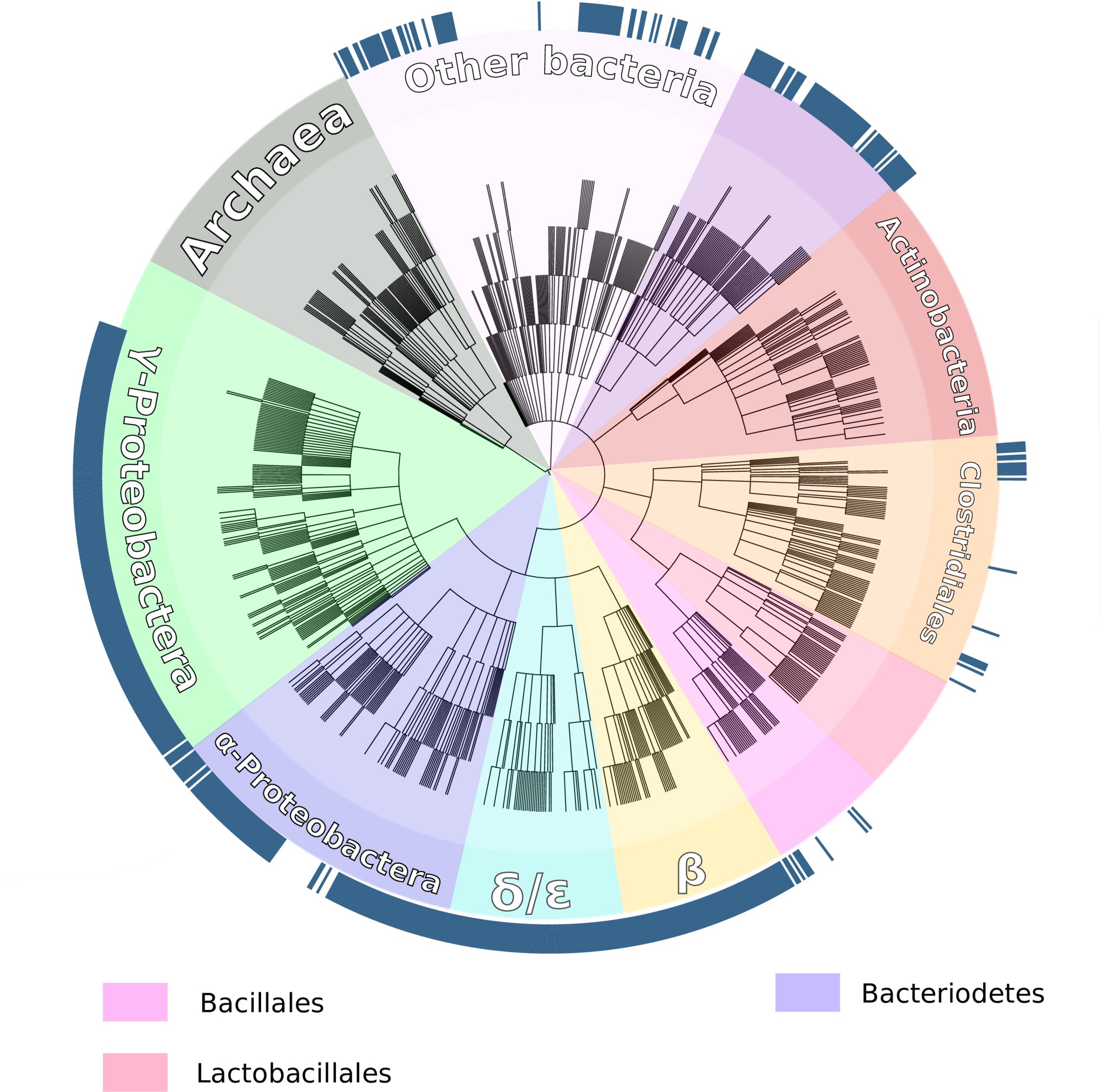

Supplement: S7 Fig — Taxonomic tree of assembled bacterial genomes from NCBI. Tree was constructed using NCBI commontree, blue bars indicate the presence of a Ton or Tol operon. (TIF) [file pcbi.1005652.s007.tif]

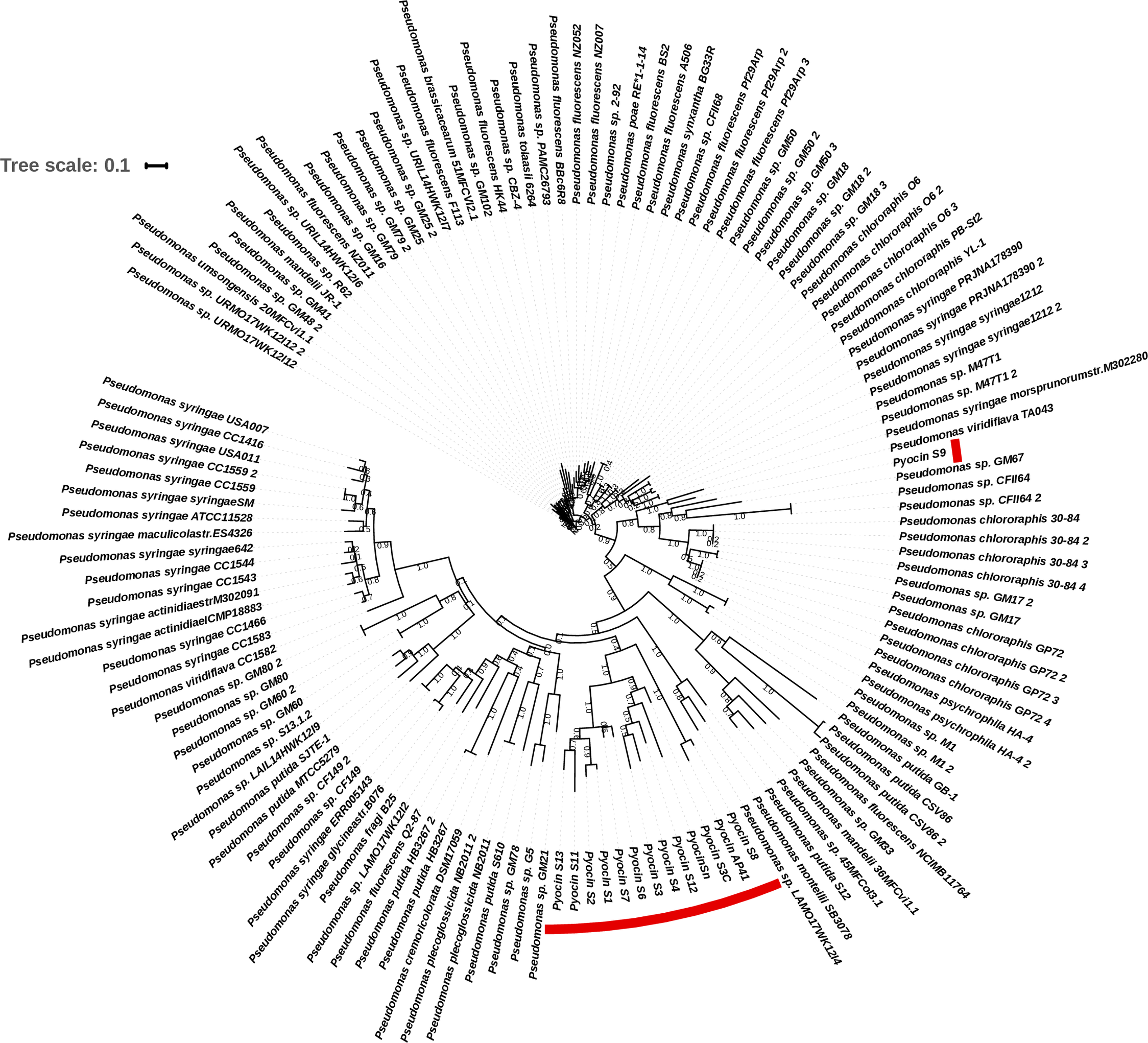

Supplement: S8 Fig — Sequences from Pseudomonas spp. were aligned using MUSCLE and trees built using Mega 6.0 using the neighbour-joining algorithm with 1000 bootstrap replicates. Red boxes indicate the position of the pyocins of P. aeruginosa. (TIF) [file pcbi.1005652.s008.tif]

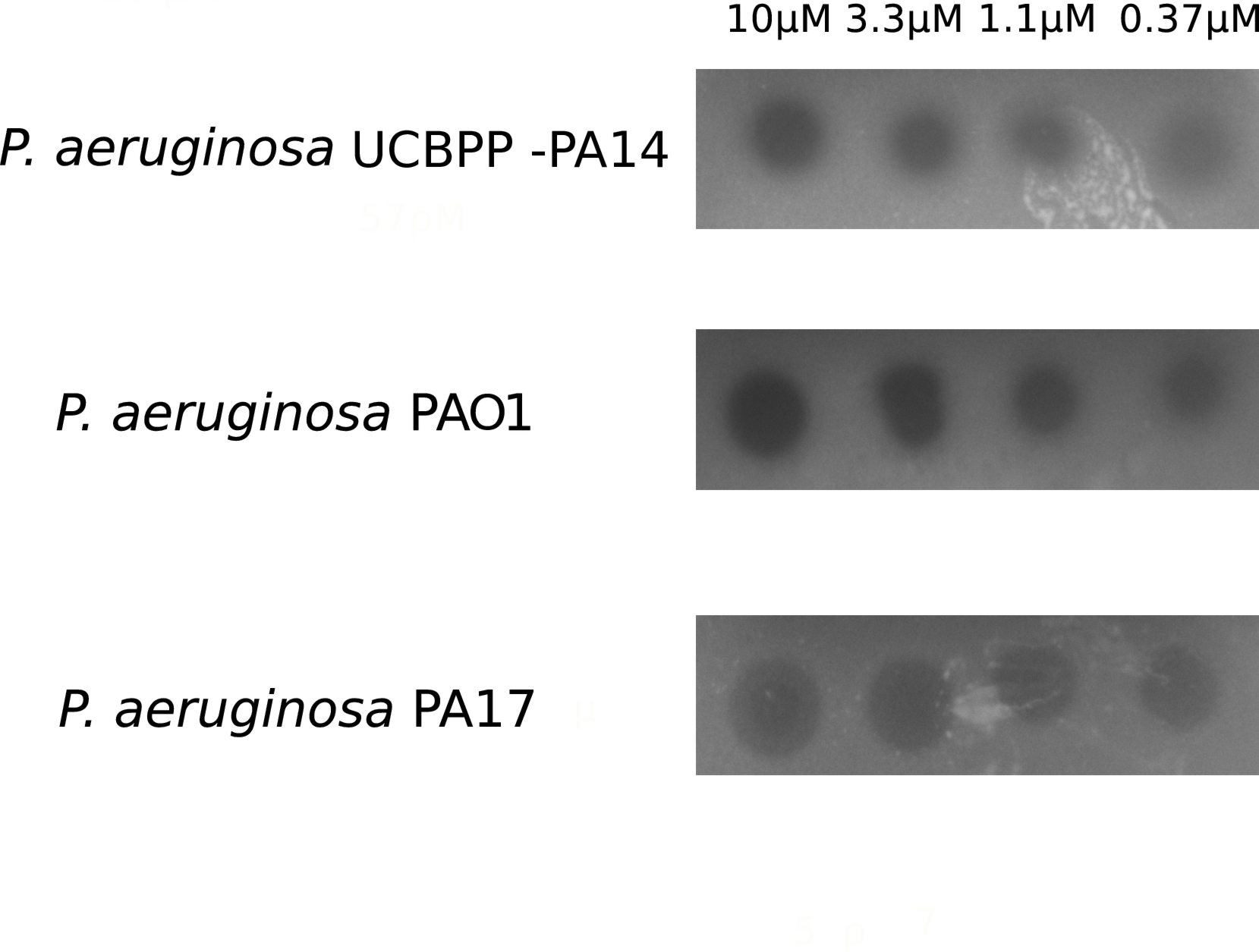

Supplement: S9 Fig — Pyocin Sn (Group VIII, Fig 3) is a non-HNH type DNase bacteriocin (88.8 kDa) newly identified by our bioinformatics analysis. As a test of the validity of this identification, pyocin Sn in combination with its immunity protein was overexpressed and purified from E. coli extracts and its activity against three strains of P. aeruginosa demonstrated. See Materials & methods for further details. (TIF) [file pcbi.1005652.s009.tif]
