# Supplementary material for: Diversity and distribution of nuclease bacteriocins in bacterial genomes revealed using Hidden Markov Models
Source: PLoS Comput Biol. 2017 Jul 17;13(7):e1005652. doi: 10.1371/journal.pcbi.1005652 (PMC5536347; doi:10.1371/journal.pcbi.1005652)
Supplement: S1 Table — PFAM profiles were first used to identify NBs. To test the robustness of our strategy, iterated profiles were created from the newly identified NBs and used in a second search. (PDF) [file pcbi.1005652.s010.pdf]

Supplementary Table 1: HMM profiles used to identify the conserved motifs of NB cytotoxic domains and associated immunity proteins.

Pfam accession	Name	Description
	DNase/IMM	
PF12629.2	Colicin-DNase	Cytotoxic domain of DNase colicins
PF01320.13	Colicin Pyocin	Colicin DNase immunity protein
	tRNase	
PF11429.3	Colicin D	tRNase cytotoxic domain of Colicin D
PF09204.5	Colicin-immun	Immunity protein for Colicin D like tRNases
PF12106.3	Colicin_C	tRNase cytotoxic domain of Colicin E5
PF11480.3	ImmE5	Immunity protein for Colicin E5 like tRNases

PF09000.5	Cytotoxic	rRNases cytotoxic domain of Colicin E3
-	ColE3_Immunity	Immunity protein for ColE3 like
		rRNases
-	Non-HNH DNase	Cytotoxic domain of Pyocin S3
-	Non-HNH DNase	Immunity protein of Pyocin S3
