# Supplementary material for: Diversity and distribution of nuclease bacteriocins in bacterial genomes revealed using Hidden Markov Models
Source: PLoS Comput Biol. 2017 Jul 17;13(7):e1005652. doi: 10.1371/journal.pcbi.1005652 (PMC5536347; doi:10.1371/journal.pcbi.1005652)
Supplement: S2 Table — Species that were identified as encoding a NB within their genome and the frequency of occurrence. (PDF) [file pcbi.1005652.s011.pdf]

Supplementary Table 2. Distribution of NBs amongst species

Species	No. in pubMLST Multispecies Database	Total NB (%)	Non-HNH	E5 tRNase	HNH	tRNase	rRNase
Citrobacter freundii	18	1 (5.6)	0 (0.0)	0 (0.0)	1 (5.6)	0 (0.0)	0 (0.0)
Edwardsiella tarda	7	1 (14.3)	0 (0.0)	0 (0.0)	1 (14.3)	0 (0.0)	1 (14.3)
Enterobacter aerogenes	51	31 (60.8)	0 (0.0)	0 (0.0)	8 (15.7)	2 (3.9)	28 (54.9)
Enterobacter cloacae	391	15 (3.8)	0 (0.0)	0 (0.0)	1 (0.3)	0 (0.0)	14 (3.6)
Erwinia tasmaniensis	1	1 (100.0)	0 (0.0)	0 (0.0)	1 (100.0)	0 (0.0)	0 (0.0)
Escherichia coli	6721	304 (4.5)	0 (0.0)	29 (0.4)	146 (2.2)	129 (1.9)	46 (0.7)
Klebsiella oxytoca	80	1 (1.3)	0 (0.0)	0 (0.0)	1 (1.3)	0 (0.0)	0 (0.0)
Klebsiella pneumoniae	1596	493 (30.9)	1 (0.1)	0 (0.0)	391 (24.5)	0 (0.0)	232 (14.5)
Klebsiella sp.	8	1 (12.5)	0 (0.0)	0 (0.0)	1 (12.5)	0 (0.0)	0 (0.0)
Morganella morganii	10	6 (60.0)	0 (0.0)	0 (0.0)	0 (0.0)	0 (0.0)	6 (60.0)
Morganella sp.	2	1 (50.0)	0 (0.0)	0 (0.0)	0 (0.0)	0 (0.0)	1 (50.0)
Pantoea sp.	9	1 (11.1)	0 (0.0)	0 (0.0)	1 (11.1)	0 (0.0)	0 (0.0)
Pectobacterium carotovorum	4	1 (25.0)	0 (0.0)	0 (0.0)	0 (0.0)	1 (25.0)	0 (0.0)
Proteus mirabilis	17	6 (35.3)	0 (0.0)	4 (23.5)	5 (29.4)	0 (0.0)	0 (0.0)
Providencia alcalifaciens	13	4 (30.8)	0 (0.0)	0 (0.0)	4 (30.8)	0 (0.0)	0 (0.0)
Providencia rettgeri	2	1 (50.0)	0 (0.0)	0 (0.0)	1 (50.0)	0 (0.0)	0 (0.0)
Pseudomonas aeruginosa	1024	874 (85.4)	198 (19.3)	162 (15.8)	440 (43.0)	286 (27.9)	22 (2.1)
Pseudomonas avellanae	2	1 (50.0)	0 (0.0)	0 (0.0)	1 (50.0)	0 (0.0)	0 (0.0)
Pseudomonas brassicacearum	2	1 (50.0)	0 (0.0)	0 (0.0)	0 (0.0)	0 (0.0)	1 (50.0)
Pseudomonas chlororaphis	5	5 (100.0)	5 (100.0)	0 (0.0)	5 (100.0)	0 (0.0)	5 (100.0)
Pseudomonas coronafaciens	1	1 (100.0)	0 (0.0)	0 (0.0)	1 (100.0)	0 (0.0)	0 (0.0)
Pseudomonas cremoricolorata	1	1 (100.0)	0 (0.0)	0 (0.0)	1 (100.0)	0 (0.0)	0 (0.0)
Pseudomonas fluorescens	26	16 (61.5)	1 (3.8)	1 (3.8)	2 (7.7)	0 (0.0)	13 (50.0)
Pseudomonas fragi	2	1 (50.0)	0 (0.0)	0 (0.0)	1 (50.0)	0 (0.0)	0 (0.0)
Pseudomonas mandelii	2	2 (100.0)	0 (0.0)	0 (0.0)	1 (50.0)	0 (0.0)	2 (100.0)
Pseudomonas monteilii	3	2 (66.7)	0 (0.0)	0 (0.0)	2 (66.7)	0 (0.0)	0 (0.0)
Pseudomonas plecoglossicida	1	1 (100.0)	1 (100.0)	0 (0.0)	1 (100.0)	0 (0.0)	0 (0.0)
Pseudomonas poae	1	1 (100.0)	0 (0.0)	0 (0.0)	0 (0.0)	0 (0.0)	1 (100.0)
Pseudomonas psychrophila	1	1 (100.0)	0 (0.0)	0 (0.0)	1 (100.0)	0 (0.0)	1 (100.0)
Pseudomonas putida	27	13 (48.1)	2 (7.4)	0 (0.0)	12 (44.4)	0 (0.0)	0 (0.0)
Pseudomonas sp.	77	43 (55.8)	0 (0.0)	1 (1.3)	27 (35.1)	0 (0.0)	26 (33.8)
Pseudomonas synxantha	1	1 (100.0)	0 (0.0)	0 (0.0)	0 (0.0)	0 (0.0)	1 (100.0)
Pseudomonas syringae	105	80 (76.2)	0 (0.0)	0 (0.0)	80 (76.2)	0 (0.0)	3 (2.9)
Pseudomonas taiwanensis	2	1 (50.0)	0 (0.0)	0 (0.0)	1 (50.0)	0 (0.0)	0 (0.0)
Pseudomonas tolaasii	3	1 (33.3)	0 (0.0)	0 (0.0)	0 (0.0)	0 (0.0)	1 (33.3)
Pseudomonas umsongensis	1	1 (100.0)	0 (0.0)	0 (0.0)	0 (0.0)	0 (0.0)	1 (100.0)
Pseudomonas viridiflava	3	2 (66.7)	0 (0.0)	0 (0.0)	1 (33.3)	0 (0.0)	1 (33.3)
Salmonella enterica	8195	24 (0.3)	0 (0.0)	0 (0.0)	22 (0.3)	1 (0.0)	2 (0.0)
Serratia marcescens	197	41 (20.8)	0 (0.0)	0 (0.0)	27 (13.7)	13 (6.6)	2 (1.0)
Serratia proteamaculans	1	1 (100.0)	0 (0.0)	0 (0.0)	0 (0.0)	1 (100.0)	0 (0.0)
Serratia sp.	8	1 (12.5)	0 (0.0)	0 (0.0)	0 (0.0)	0 (0.0)	1 (12.5)
Shigella boydii	171	30 (17.5)	0 (0.0)	1 (0.6)	0 (0.0)	29 (17.0)	0 (0.0)
Shigella flexneri	518	2 (0.4)	0 (0.0)	0 (0.0)	1 (0.2)	1 (0.2)	0 (0.0)
Shigella sonnei	675	322 (47.7)	0 (0.0)	168 (24.9)	113 (16.7)	1 (0.1)	43 (6.4)
Xenorhabdus nematophila	3	1 (33.3)	0 (0.0)	0 (0.0)	0 (0.0)	0 (0.0)	1 (33.3)

Yersinia bercovieri	3	1 (33.3)	0 (0.0)	0 (0.0)	1 (33.3)	0 (0.0)	0 (0.0)
Yersinia enterocolitica	115	92 (80.0)	0 (0.0)	0 (0.0)	64 (55.7)	0 (0.0)	28 (24.3)
Yersinia frederiksenii	14	2 (14.3)	0 (0.0)	0 (0.0)	2 (14.3)	0 (0.0)	0 (0.0)
Yersinia intermedia	14	4 (28.6)	0 (0.0)	0 (0.0)	4 (28.6)	0 (0.0)	0 (0.0)
Yersinia kristensenii	8	5 (62.5)	0 (0.0)	0 (0.0)	5 (62.5)	0 (0.0)	0 (0.0)
Yersinia mollaretii	11	11 (100.0)	0 (0.0)	0 (0.0)	11 (100.0)	0 (0.0)	1 (9.1)
Yersinia pseudotuberculosis	44	25 (56.8)	0 (0.0)	0 (0.0)	25 (56.8)	0 (0.0)	0 (0.0)
Yersinia sp.	4	2 (50.0)	0 (0.0)	0 (0.0)	2 (50.0)	0 (0.0)	0 (0.0)
