# Supplementary material for: Diversity and distribution of nuclease bacteriocins in bacterial genomes revealed using Hidden Markov Models
Source: PLoS Comput Biol. 2017 Jul 17;13(7):e1005652. doi: 10.1371/journal.pcbi.1005652 (PMC5536347; doi:10.1371/journal.pcbi.1005652)
Supplement: S3 Table — Tol was differentiated from Ton by the presence of a PD40 repeat that is part of the TolB protein. (PDF) [file pcbi.1005652.s012.pdf]

Supplementary Table 3. HMM profiles used to identify the conserved regions of proteins in the Tol/Ton operons.

PFAM accession	Name	Description
PF03544.9	TonB C	C-terminal region of TonB. Interacts with TonB box.
PF01618.11	MotA ExbB	MotA/TolQ/ExbB proton channel family
PF02472.11	ExbD	Biopolymer transport protein ExbD/TolR/MotB
PF07676.7	PD40	WD40-like Beta Propeller Repeat associated with TolB
